# The Experiences of Adolescents and Young Adults with Digital Supportive Care Interventions for Cancer: A Systematic Review of Qualitative Studies

**DOI:** 10.3390/cancers17050736

**Published:** 2025-02-21

**Authors:** Mashiad Mostafa, Y. Sarah Chae, Kelcey A. Bland, Helen McTaggart-Cowan

**Affiliations:** 1 Cancer Control Research, BC Cancer Research Institute, Vancouver, BC V5Z 1L3, Canada; mmostafa@bccrc.ca (M.M.); schae@bccrc.ca (Y.S.C.); kelcey.bland@ubc.ca (K.A.B.); 2 Faculty of Health Sciences, Simon Fraser University, Burnaby, BC V5A 1S6, Canada; 3 Department of Physical Therapy, University of British Columbia, Vancouver, BC V6T 1Z3, Canada

**Keywords:** cancer, supportive care, digital, technology, adolescents, young adults

## Abstract

**Background:** Evidence suggests the importance of cancer supportive care for adolescents and young adults (AYAs), and digital technology may provide tailored care that is flexible, affordable and accessible. However, AYAs’ experiences with these digital cancer supportive care interventions are currently unclear. **Objective:** The aim of this review is to systematically identify and explore potential intervention facilitators, barriers and areas of improvement. **Methods:** We conducted a comprehensive search of MEDLINE (Ovid), EMBASE, PsycINFO and CINAHL for mixed methods and qualitative studies, published between 2000 and 2023, focusing on the experiences of AYAs between the ages of 15 and 39 years using digital supportive care interventions for cancer. Studies involving only pediatric and older populations were excluded. The identified studies were critically appraised and thematically analyzed. **Results:** Twenty-three digital interventions were identified. They varied in modality and addressed different aspects of supportive care (e.g., physical activity, psychological well-being and symptom management). Participants’ experiences with the intervention attributes (e.g., appropriate content, flexible choices, seamless technology and inclusive environment) influenced their physical and psychological health, connections and communication skills, and autonomy. **Conclusions:** Overall, AYAs reported favorable experiences with digital interventions when provided with tailored supportive care for cancer. Digital interventions may help to increase reach and access to supportive care for cancer; however, barriers to delivery, such as faulty technology or cumbersome intervention features, can negatively impact participant experiences and may reduce engagement.

## Simple Summary

Adolescents and young adults (AYAs) with cancers between the ages of 15 and 39 years face health challenges affecting their quality of life during treatment and beyond. Cancer supportive care aims to improve the patient’s overall health and well-being. The digitalization of supportive care may increase accessibility, reduce out-of-pocket costs and provide specialized services. However, there is limited knowledge of how acceptable these inventions are from the patient’s perspective. In our systematic review of qualitative studies, we identified 23 studies exploring AYAs’ experiences with digital cancer care interventions, exploring potential intervention facilitators, barriers and areas of improvement. Findings suggest that experiences with the intervention attributes (e.g., appropriateness of the content, flexibility of the choices, seamlessness of the technology and inclusivity of the environment) may either facilitate or hinder the participant’s physical and psychological health, connections and communication building, and autonomy.

## 1. Introduction

Cancers that affect adolescents and young adults (AYA) are a significant global burden, with 1.19 million new AYA cancer cases in 2019 [1]. AYAs are a heterogeneous population with a broad age range of 15–39 years [2,3,4]. While there is a general consensus that children are defined as 0–14 years, the upper age limit of 39 years for young adults is intended to capture the intersectionality of epidemiological, clinical, biological and psychosocial features that make cancer in AYAs a unique disease constellation [5]. For many AYAs, both the cancer diagnosis and subsequent treatments can lead to short- and long-term adverse effects, such as changes in cognitive functioning and new chronic and mental health conditions [6,7] extending into their later lives and disrupting the achievement of milestones.

The provision of tailored supportive care may improve the health and well-being of AYAs with cancer. Supportive care is the prevention and management of adverse effects of cancer and its treatment [8]. It encompasses but is not limited to exercise and rehabilitation, nutrition, psychological health, sexual health, and spiritual health. There has been considerable development in supportive care to promote holistic patient-centred cancer care [9,10]. Research has shown that physical activities, paired with nutritional and rehabilitation plans, alleviate the adverse effects of cancer and its treatment among AYAs by improving functional capacity, managing symptoms and optimizing treatment outcomes [8]. Counselling and support groups can address anxiety, depression, post-traumatic stress disorder and readjustment disorder [11,12].

Since the COVID-19 pandemic, healthcare services have seen an increase in digitalization [13]. These digital services can be in the form of mobile health, text messages, software applications (i.e., “apps”), wearable devices, and telehealth/telemedicine. Digital technology has the potential to provide tailored, flexible, and cost-effective supportive care, as well as eliminate barriers to access for individuals not residing in urban areas [14,15]. These factors are advantageous, considering many AYAs have access to smartphones and the internet [16]. Further, AYAs have expressed interest in digital interventions, especially for age-appropriate psychosocial and peer support [17,18].

Most existing work primarily evaluates the usability of digital supportive care interventions [19,20,21]. While the information generated from usability studies is advantageous in guiding the design of the interventions, there is limited information about the experiences of AYAs with cancer regarding these digital supportive care interventions. Understanding the experiences of AYAs can help improve digital intervention development and delivery to prioritize patient needs and access to supportive care. Recently, a review was conducted that systematically captured pediatric cancer survivors’ user experiences with digital health interventions [22]. The authors found that digital interventions were deemed to be acceptable and convenient for delivering care to the pediatric oncology population across the cancer care continuum. However, the perceptions and needs of the AYA cancer population are not comparable as the life transitions, such as gaining independence, navigating education, and developing relationships, are distinct from the pediatric cohort, as well as the older population. The AYAs’ unique perceptions and distinctive needs shape their experiences with digital supportive care interventions for cancer. This necessitates a systematic assessment of the experiences of AYAs with cancer with digital supportive care. In this work, we conducted a systematic review of qualitative studies exploring experiences with digital supportive care interventions among AYAs with cancer. Our systematic review aimed to thematically highlight the perceived intervention barriers, facilitators, and areas of improvement for wider programmatic implementation in digital supportive care interventions.

## 2. Methods

This systematic review is registered in the PROSPERO database (CRD42024485898).

### 2.1. Information Sources and Search Strategy

A comprehensive search of MEDLINE (Ovid), PsycINFO, CINAHL, and Embase databases was conducted. The search strategy was developed in consultation with research librarian at the BC Cancer Research Institute. Search results were restricted to the English language from 1 January 2000 to 31 December 2023, in line with the emergence of digital intervention for AYA supportive care for cancer. The literature search was carried out from 6 November 2023 to 31 December 2023. Example search terms include “cancer/neoplasm”, “adolescents and young adults”, “supportive care”, “digital”, or “technology”, and “qualitative analysis”. The comprehensive search strategies are available in Appendix A (Table A1) and as Supplementary Materials (Tables S1–S3).

### 2.2. Inclusion and Exclusion Criteria

For a study to be eligible for inclusion, the following criteria needed to be fulfilled: (1) focused on digital supportive care interventions (e.g., mobile applications, e-health platforms, and wearable devices); (2) sampled participants between the ages of 15 and 39 years who had received a diagnosis of cancer, and were awaiting treatment, receiving treatment or completed treatment; and (3) designed to collect data on participants’ experiences with the digital intervention using qualitative or mixed method approaches. If the outer age bounds of the participants extended beyond the AYA age range, the study was considered for inclusion if the reported mean or median was within the AYA age range. Descriptive qualitative studies and mixed methods studies with a qualitative component were selected to capture richer insights into participants’ experiences with digital supportive care interventions; these insights were illustrated through verbatim quotations.

Studies were excluded if they: (1) were empirical in nature without participant quotations presented, including quantitative studies; (2) involved only pediatric (<15 years) and older populations (>40 years); (3) included participants outside the AYA age range; (4) were need assessment studies to inform the design of digital supportive care intervention rather than collecting experiences; and (5) were review papers or gray literature including commentaries, and conference abstracts. The decision to exclude empirical studies, reviews and gray literature without participant quotations was made to gain a qualitative understanding of the nuances of participants’ experiences with the digital interventions.

### 2.3. Study Selection

All authors independently reviewed article eligibility using Rayyan [23], an online software system. At least two authors independently screened titles and abstracts and reviewed the full-text versions of the articles to determine eligibility. Discrepancies were discussed among all authors until consensus was reached. Preferred Reporting Items for Systematic Reviews and Meta-Analyses (PRISMA) [24] statement was used to design and summarize the identified studies in this systematic review. The comprehensive PRISMA 2020 checklist is available as a Supplementary Material (Table S4).

### 2.4. Data Extraction

The extracted information included authors, year, country, study design, supportive care type (e.g., psychosocial, physical activity), digital health intervention type (e.g., facilitated virtual programs, self-directed websites, software applications, and peer support systems), participant characteristics (e.g., age, cancer type), sample size, qualitative data collection mode, and qualitative analytic method. For mixed methods studies, only the sample size of the qualitative phase is reported. Two authors (M.M. and Y.S.C.) completed data extraction in duplicate, with a third author (H.M.C.) randomly extracting data for 30% of articles to ensure consistency.

### 2.5. Data Analysis and Quality Assessments

All qualitative data were imported to the NVivo version 14 [25] for coding and synthesis. We conducted a thematic synthesis [26], which involved familiarizing data, developing a coding framework (Table S5), and identifying themes. Two authors (M.M. and Y.S.C.) independently coded all presented participant quotes and all data in the results section line-by-line; content in the discussion section was not coded. Team discussions addressed any coding discrepancies that arose. The Critical Appraisal Skill Programme (CASP) checklist [27] for qualitative research was used to assess the quality of the included articles, and the results are available as Supplementary Material (Table S6). The checklist contains ten criteria for research validation, methods, risk of bias and research impact. The first two authors independently assessed all studies in duplicate, and the fourth author randomly assessed 30% of articles to ensure accuracy. Confidence in the Evidence from Reviews of Qualitative Research (GRADE-CERQual) [28] approach was utilized to assess the confidence in the synthesized evidence. The framework consists of four elements: methodological limitations, coherence, adequacy, and relevance. The first author independently assessed each of the themes and reviewed by another author (H.M.C.) to ensure consistency. The comprehensive framework is available as Supplementary Material (Table S7).

## 3. Results

### 3.1. Intervention Characteristics

**Figure 1:**
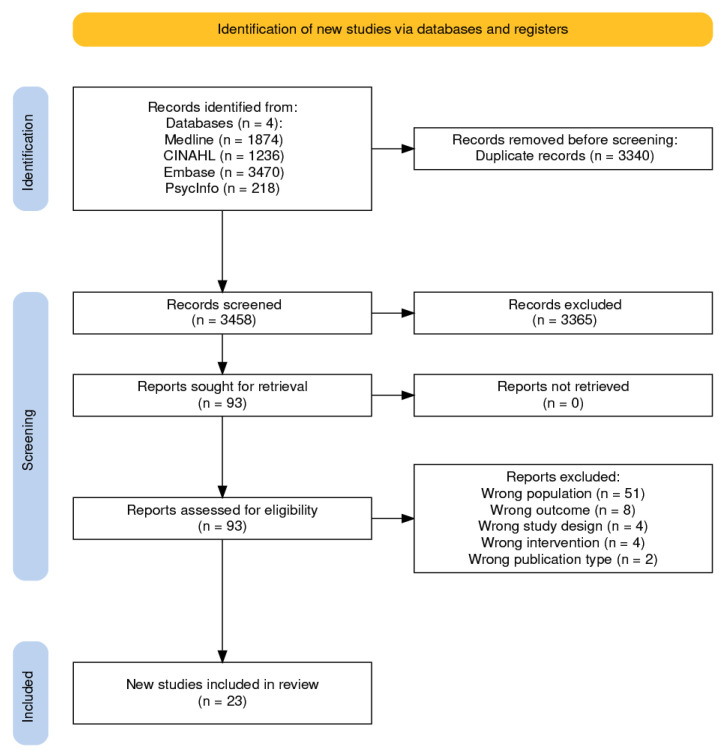
PRISMA2020-Compliant flow diagram [29].

In total, 6798 studies were identified; after removing duplicates, 3458 studies were screened. Twenty-three studies containing 21 interventions and 581 patient participants met the eligibility criteria for inclusion in the review (Figure 1).

The majority of the interventions (*n* = 13) aimed to provide psychosocial supportive care [30,31,32,33,34,35,36,37,38,39,40,41,42,43]. Three interventions focused on physical activity-related supportive care [44,45,46] and two on symptom management [47,48]. The remaining three interventions were multidimensional [49,50,51,52], addressing more than one supportive care type. Table 1 summarizes the characteristics of the included studies.

The delivery modes of the digital interventions were: (1) facilitated virtual programs involving synchronous or asynchronous virtual content through emails and teleconference platforms (e.g., recorded classes); (2) self-directed websites in an interface with the purpose of an information source (e.g., modules, recorded videos and forums); (3) software applications (i.e., apps), developed as downloadable programs and require compatibility with phones and tablets (e.g., wearable activity trackers are often paired with activity-based apps); and (4) peer support system providing standalone peer interactions through forums or communication channels. In our review, eight interventions were facilitated virtual programs [36,37,38,41,43,44,50,51,52]; four (each intervention resulted in two studies) were delivered as self-directed websites [30,32,33,39,40]; four were designed as software applications [35,42,47,48], and one intervention [34] was a peer support system. Four interventions were delivered through more than one mode [31,45,46,49].

Nineteen digital interventions were specifically designed for individuals with cancer in the AYA age range. While most interventions enabled participation across mixed cancer types (*n* = 18), five were specific to testicular [30,49], breast [32,33], or sarcoma cancers [31]. Only one intervention was designed for non-metastatic patients [32,33], while the majority did not differentiate across cancer stages. Further, 12 interventions were inclusive of participants across all cancer phases (i.e., diagnosis, treatment, survivorship) [31,32,33,35,36,37,38,39,40,42,44,48,52], while nine interventions focused on one specific phase within the cancer care continuum: diagnosis [30], active treatment [41,47], or survivorship [34,43,45,46,49,50,51]. Thirteen interventions embedded two-way communication in their designs, which enabled communication between the participants and the facilitator or affiliated healthcare providers (HCPs) [30,31,34,37,38,39,42,43,44,45,46,48,50,51].

The identified studies employed either mixed methods (*n* = 17) or qualitative (*n* = 6) designs, with sample sizes ranging from six [49] to 83 participants [47]. Participants’ experiences with the digital intervention were elicited using open-ended questionnaires (*n* = 13) or semi-structured interviews (*n* = 17). Thematic analysis (*n* = 15) was the most frequently used technique for data synthesis. 

#### Quality Assessments

The CASP checklist (Table S6) was used to assess the qualitative component of the studies. The authors (M.M. and Y.S.C.) ranked the eligible studies as robust (*n* = 2), moderately strong (*n* = 8), moderate (*n* = 9), or weak (*n* = 4) based on how many of the ten CASP criteria were met. Most of the eligible studies failed to meet the criteria of appropriate research design (*n* = 14) and adequate examination of biases between the researchers and the participants (*n* = 15).

We assessed the confidence of the 14 generated themes (Table 2) using the GRADE-CERQual assessment framework. The confidence is high in three of the themes, moderate in ten, and low in only one theme (Table S7). The main limitations of our synthesized findings stem from studies not justifying or clarifying their methodologies, with some concerns about the adequacy and coherence of the secondary data collected from these published studies. 

### 3.2. Experiences with Digital Interventions

**Figure 2:**
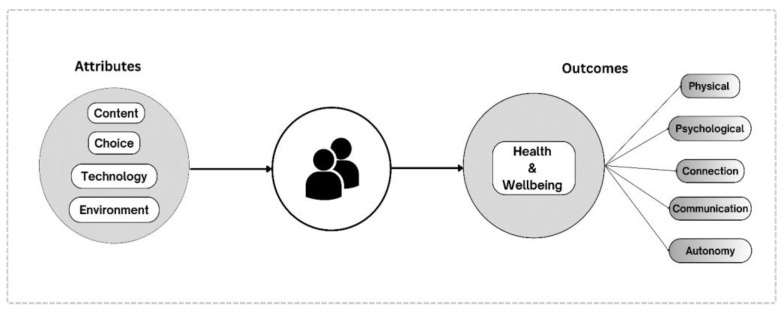
Conceptual Framework—AYAs’ Experiences with Digital Cancer Supportive Care.

AYAs with cancer exhibited diverse experiences and outcomes when using digital supportive care interventions. The digital interventions contained specific attributes that aid in participant experiences through content appropriateness, choice availability, technological usability, and environmental inclusiveness. We conceptualized a framework illustrating the relationship between the experiences with these embedded attributes and the outcomes gained from the interventions (Figure 2). A positive experience with the attributes may lead to positive outcomes for the AYAs with cancer, ranging from an improvement in their health, enhancement in their connections and communication skills to a gain in autonomy. Table 2 contains a summary of the identified themes and exemplary quotes.

#### Intervention Attributes

AYAs with cancer desired age-appropriate and relevant digital information. Positive experiences were associated with AYAs’ comprehension and receptivity to the content of the intervention. This was especially pertinent to self-directed websites, including images, videos, and survivor testimonials [30,31,32,33,34,35,36]. Participants did not appreciate busy layouts and “*impersonal*” content regardless of its delivery mode. Participants’ experiences were suboptimal when the content of the interventions did not align with their cancer type, stage, or specific needs [30,35,37,39,42,49].

*[I am] two years post-surgery [so] the course was not as relevant to me. However, I definitely think it would be very useful to have available to people coping with chemo and cancer.*

(Heiniger et al. (2017) [49]|Multidimensional Self-Directed Information Module)

Some interventions provided participants with the choice to spend “*more time on [aspects of the intervention] that [was] more relevant [to them]*” [49]. By having the option to personalize the interventions, participants can adapt the interventions to meet their self-identified needs. Examples of personalization include options to select convenient dates and times for activity-related interventions [30,40,49,51] and to limit the notifications when participants do not wish to receive multiple alerts throughout the day or week [31,34,37,39,43,48]. Participants with compromised immune systems [52] and other co-morbidities especially appreciated the “*convenience of being able to participate [in the intervention] with low blood counts and not having to drive anywhere*” [38].

AYAs preferred supportive care interventions when the technology was “*fairly intuitive*” and easy to use and navigate [32,37,38,40,42,46,48,49]. Technological barriers—software glitches, time-outs, and battery issues—were observed to jeopardize the overall usability of the intervention [32,33,43,44].

*[The activity tracker] was not wanting to keep track of the distance I was walking very well, and it wasn’t always syncing very well necessarily.*

(Mendoza et al. (2017) [45]|Multimodal Physical Activity Intervention)

AYAs with cancer expressed their preference for environments that they perceived to be inclusive. For some, favourable environments promoted psychological safety and enabled the AYAs to share their anxieties and thoughts with their peers. This was facilitated by ensuring anonymity and inclusivity among participants, for example, support groups exclusively for invited members [35,40,41,43,48]. AYAs attributed safety to the presence of a facilitator who “*conveyed patience, respect, and understanding*”, fostering a comfortable, active, and inclusive environment [50,51,52].

*I think just the personality of the instructor, they just created a safe and easy space [to practice yoga], it felt comfortable right away and non-threatening and there was never any pressure.*

(Wurz et al. (2023) [52]|Multidimensional Facilitated Virtual Behaviour Change Program)

**Table 2:** Descriptive themes and exemplary quotes.

Experiences That Enhance Outcomes		
	Themes	Quotes
Intervention Attributes	Appropriate Content	“I’d say definitely those videos, it just sort of put a human touch on the whole situation...” [30] “…tailored exactly to what I needed” [44] “It was engaging because of the [educational] videos [and] … to hear it from other people who’ve gone through it and their experiences and the stuff that a doctor can’t tell you—I found that really terrific”. [49]
	Flexible Choice	“We were able to complete the exercises on our own schedule”. [33] “…There was stuff you could try at home and like do yourself. So I liked that”. [48] “[you could] pick up what you want and what you need from the website”. [49]
	Seamless Technology	“The smartphone app is pretty handy to know if you’re on track for your goals. seeing the trends of your activity. being able to join other groups. compete against people”. [45] “It was really easy. It was very straightforward. It wasn’t really complicated. It was just like simplified so it was easy to use for little kids”. [48] “User-friendly”. [49]
	Inclusive Environment	“I liked that it was a safe place to expose your own thoughts/anxieties” [31] “Easy to access information within privacy of own home…” [40] “It was a free space and I felt very open, cancer was normal in that space, which is rare in my day-to-day life, where I feel like I walk around with a heavy little secret sometimes”. [43]
Intervention Outcome: Health and Well-being	Improved Physical Health	“New burst of energy”; [44] “I feel energised and alive”. “I thought the pain help ideas were really awesome. When they suggested like different things that you could do? Those were really helpful”. [48]
	Improved Psychological Health	“I did not feel so alone in the situation, it reassured me that okay, it is normal to feel like that, others have also experienced it and their trajectory has also been like that, so I’m not a single weird case”. [35] “I’ve mostly just used the coping mechanisms....A lot of the questions I felt calmed me down so that worked for me. They were pretty well done”. [42] “Allowed me to remain positive”; “release anxieties”. [44]
	Enhanced Connections and Communication Skills	“…[The website] gave me the proper questions that I need to ask, not only the oncologist, but also the nurses when I went into chemo”. [30] “My husband and I are feeling more connected to one another. We have learned to communicate and express ourselves better”. [33] “[Group was] life-changing for me. I can’t express how important it is for me to be able to talk to people who went through things like I did, as I never got to meet anyone while I was in the hospital and always felt so isolated”. [36] “These hearts that you would get, somehow they make you feel supported, that you’re not alone in this/…/. [the likes and comments in the discussion forum] even made me feel that I would have liked to meet these girls, it reached a level of some kind of affinity”. [39] “I think the biggest thing of the whole intervention is actually having you [the facilitator] at the other end. To have a person to actually talk to”. [41] “[The program] gave some ideas on how to talk about problems and to get my family to “open up”. [43] “Basically it helped be a visual for me and my doctor to help talk about it because right there open on the table and we discussed it and came up with a plan”. [47] “My kids, their support was great because they started eating everything that I was eating so to see that I wasn’t doing it alone was really good”. [51]
	Autonomy	“It gives an awareness that you have to get through no matter how hard it is at times. Some have been through something worse, and they have managed it, so even though it is not the same thing we have been exposed to, it gave peace to know that you can get through it and get well on the other side and get started in life again”. [35] “I am taking the steps to get help and more of getting more educated. That is what I am doing. I can be better…It made me think about what I can do or what I can take to get that energy and the things that help me. It did help and gave a push. I always wanted to be better after I was done because I don’t want it to come back”. [37] “You have to start somewhere. this is a good starting place. got that sense of community, that sense of accountability. just starting to move, kind of embracing life gain”. [46] “Really in the middle of the whole thing, I went ‘woo, you need to stop telling yourself that you can’t do something and continuously tell yourself that you can do something’. So, my new thing is ‘get up, get going, because you can do this”. [51]
**Experiences that Hinder Outcomes**		
Intervention Attributes	Generic Content	“Articles that don’t just pertain to emotional dealings but pragmatic tips for dealing with nausea or hair loss. Something similar to how magazines give tips and tricks since that is a very palatable format to many people that I know, and something that I really missed from a lot of coping and support forums that I’ve encountered for cancer”. [31] “It’s helpful as a survivor but isn’t very tailored to my specific worries and thoughts”. [34] “In relation to my disease, I could not... it is very rare, so I could not really see myself reflected in so much of it that was written in there”. [35] “It was the same exact wording in the message every single time, so it almost seemed like robotic”. [46] “…two years post- surgery the course was not as relevant to me. However I definitely think it would be very useful to have available to people coping with chemo and cancer”. [49]
	Limited Choices	“That there were questions every day, it was a little hard to keep up with responding” [31] “We have very limited free time available and found it difficult to finish the lessons within a week, particularly the ones that needed to be started immediately…In the end it was a bit disruptive to our normal routine”. [33] “I like you [chatbot] a lot but wish there were a few more options”; [34] “It’s [Facebook is] kind of more for older people nowadays, I don’t really find anything that would have any interest in me. Like, I would check it every two weeks or so maybe” “Snapchat would be cool for Fitbit. You could, like, Snap your Fitbit…” “I think Instagram would work well because it’s more interactive with multiple people” [45] “…Because sometimes people don’t want to like keep...um...doing the same thing over again...22 questions every time”. [48]
	Faulty Technology	“It [activity tracker] was not wanting to keep track of the distance I was walking very well, and it wasn’t always syncing very well necessarily”. [45] “I did have to charge mine every night, it didn’t last more than one day for me”. [46] “Even if I did do my case [pain assessment], it would still just keep on giving notifications. And I know that after you say, “yes” to the case, [the app will] follow-up [on the severity of pain one hour later]. But even if I would do the follow-ups, it would just keep on giving more and more [notifications]”. [48]
	Unfavorable Environment	“I would recommend it, but also something slightly less faceless if that makes sense”. [35] “So, we were quarantining with my in-laws and there was nowhere really private to go and it felt like kind of a private thing”. [41] “Facebook wasn’t especially appealing [because] the elephant in the room was that the main thing we all had in common was that we’d had cancer in the past, and that’s a weird commonality to have with strangers. it felt unaddressed”. [46]
Intervention Outcomes: Health and Well-being	Lack of Connection and Communication	“…some mental health support would have been good on the website…even if it was just like maybe a link or something like that to like—a support group…a psychologist or who to talk to…” [30] “It was difficult to actually get to know people since most of the questions or discussion topics were more “fun fact” than really getting to know. Maybe having some more personalized chat features or encouragement to post not just on prompts but on our own would also be good”. [31] “It matters that people are anonymous, then the community will not be as strong”. [35] “If there was something face-to-face with other people, that’s also a great way of getting connections and hearing what other people use for mindfulness”. [40] “The part where you get the advice from the nurse was good but then sometimes I would just miss her if I was out or my phone was on silent. So, it might be better if she left you a [text] message so that you could check what she was telling you to do”. [48] “I didn’t really feel like the sense of community [was something] that I wanted with the other participants. [Since] it was optional to have the camera on, and also I just had my screen on like speaker mode […]”. [52]
	Triggering	“…..[intervention module] the reflection often caused me to start over analyzing and I wasn’t really able to get out of it”. [31] “So, every time I opened the app, I thought ‘no, I also have to see what something else happens,’ and then things appeared I was not happy about. It was like I had to distance myself from the app to distance myself from this community”. [35] “[Not want to] spend too long wracking my own brains of that about things [sic]… it’s very difficult to disentangle …” [49]

### 3.3. Intervention Outcomes: Health and Well-Being

The experiences that AYAs with cancer have with the intervention attributes influence their overall outcome gain or loss. This outcome may reflect their overall health and well-being in terms of physical and psychological health, connection and communication building, and autonomy.

#### 3.3.1. Physical Health

Three of the 21 interventions solely focused on improving participants’ physical health [44,45,46], and all three multidimensional interventions incorporated elements of physical activity [49,50,51,52]. The provision of choice facilitated the set-up of personal goals and tracking of fitness metrics (e.g., sleep, activities, and calories). An inclusive environment was created through friendly competitions and reward systems that motivated participants to “*get out and exercise*” and establish healthy exercise and nutrition routines [45,46,47,48].

*“It actually did get me from just sitting there and go do things, go walk around or go to the gym for a half hour”.*

(Miropolsky et al. (2020) [46]|Multi-modal Physical Activity Intervention)

Moreover, their improved physical health positively extended to other dimensions of their health and “*[their] day-to-day activities*” [44]. Further, some psychosocial supportive care interventions would indirectly contribute to the improvement of AYAs’ physical health. For instance, personalized care plans [37] provided adequate content in guiding AYAs toward resources and strategies for boosting their energy and overall physical health.

#### 3.3.2. Psychological Health

Overall, AYAs’ experiences with the psychosocial supportive care interventions were positive [30,31,32,33,34,35,36,37,38,39,40,41,42,43]. Improvements in their psychological health were observed, especially when the interventions contained appropriate content addressing positive psychology, such as cognitive, behavioral and mindfulness practices, music therapies, and coping approaches. The multidimensional interventions [49,50,51,52] embedded certain elements—yoga, psychoeducational material, and autonomy support by the facilitator—to address participants’ psychological needs. The integration of peer support, either one-to-one or group, [30,31,34,35,36,38,39,41,42,52] promoted inclusion and alleviated feelings of isolation. The availability of certain choices, even the simpler ones, such as adjusting the music list or selecting class time, inspired participants to continue engaging with the intervention [52]. The provision of adequate and relevant information within the intervention, AYAs experienced a reduction in stress and anxiety:

*[…] The website was good because when I was about to start to get a bit anxious so that like, “Oh, God, what if, what if, what ifs,” I could read the information to just reassure myself, I guess, with the general facts […].*

(Conduit et al. (2022) [30]|Self-Directed Psychosocial Information Source)

While the majority of the interventions were patient-focused, there was one dyadic intervention for AYAs with breast cancer and their partners to reflect and understand each other’s psychological state and overall well-being [32,33]. In one activity, couples were able to illustrate and convey their emotional struggles and perceived hardships using metaphors. This offered them a “new perspective” of themselves and their relationships, and they learned to appreciate the gains and adjust to changes brought about by breast cancer. Some AYAs with cancer appreciated direct nurse referrals or communications built into the intervention feature [33,37,48]. They perceived the nurses’ involvement, even if not accessed, as an extra layer of psychological support in symptom management [48].

Though most intervention attributes enhanced the psychological well-being of the AYAs, some proved to be counterproductive in intervention participation. Most AYAs with cancer preferred information-based interventions, yet some experienced stressful emotions and felt overwhelmed by “*…all sorts of gloomy things*” brought forth by too much information. [35,37] Some intervention modules, such as cognitive tasks, resulted in participants to “*overanalyze*” [31] and triggering thoughts that were “*…difficult to disentangle*” [49]. Some AYAs with cancer found it stressful to coordinate the virtual sessions and expressed having difficulty finding privacy within their homes. This hindered their ability to engage fully with the intervention and achieve optimal psychological well-being [41].

Not being able to meet exercise goals in physical activity-based interventions would demotivate participants, resulting in feelings of disappointment, stress and depression [46]. Further, experiencing inappropriate content and technical challenges contributed to the low participation throughout the interventions [33,34,43]. For instance, one intervention on care plans had no instructional resources or guidelines on embedded hyperlinks, resulting in almost half the participants not utilizing the mental health resources or partaking in the intervention [37].

#### 3.3.3. Enhancing Connections and Communication

Participants valued digital supportive care interventions that promoted a sense of community through their design elements; some examples include support groups, peer videos, and communication forums [31,32,34,35,38,43,44,47,48,49,50,51]. Age- and cancer-appropriate content facilitated connection building with peers and family members, further enhancing well-being; for instance, participants felt they could relate to real-life survivor stories [49]. For many participants, confiding and connecting with peers of similar ages in a safe environment created by the intervention was a significant experience [31,35,36,45]. Participants expressed that these peer social groups were different from those typically encountered in hospitals and cancer care facilities. AYA-specific social forums were deemed important as a “*community of age*” [35] and “*… something major that is missing for the emotional side of the treatment*” [31]. These forums facilitated open discussions, and AYAs with cancer felt “*affinity*” with like-minded people [39]:

*My old friends […] do not know what [cancer is like] because they have not felt it on their own body, so it is nice to talk to someone who knows the special fatigue that you can feel, which is not just a fatigue you can sleep away. Old friends can’t talk about hair loss and wigs, they do not understand.*

(Hanghøj et al. (2023) [35]|Psychosocial Social App and Forum)

Some digital interventions also facilitated connections with family and friends. Couple-specific interventions encouraged participants to have open dialogues to foster their relationships and give them a better perspective of each other [41,42]. Programs on building peer networks (e.g., the buddy system) enabled AYAs with cancer to strengthen their bonds with friends, families, and even acquaintances [41,46,50,51]. Enhancing connections motivated the participant to continue participating in the intervention to further their learnings and interactions.

Moderators, such as health coaches and instructors, were also integral to some of the interventions by promoting inclusivity [37,39,40,41,42,47,52]. They fostered an environment that was “*welcoming of individuals with different cancer diagnoses and abilities*” [52], allowing AYAs with cancer to communicate, connect and celebrate milestones together. Further, some interventions indirectly contributed to the AYAs’ confidence when interacting with their HCPs. For instance, comprehensive testicular cancer content on a website [30] enabled participants to identify questions for their HCPs at their medical appointments. A symptom management intervention [47] functioned as a visual cue for participants to prioritize their symptoms and to effectively communicate with their HCPs:

*Everything is laid out and just put forward, so you can get right talking about what is the biggest problem.*

(Erickson et al. (2019) [47]|Symptom Management Software Application)

While the ability to improve communication and connection was important for many AYAs with cancer, some participants were not able to engage their families to participate in interventions or form connections with the peer groups [39,41,42]. This may be a result of choices embedded in the design of the interventions [31,37,39,40,49,50,51]. For example, while the camera function aligns with some AYAs’ preferences for anonymous interactions, some associated anonymity with ingenuity and desired more face-to-face interaction [31,47] among participants and/or healthcare providers:

*[…] I always turn my camera off, pretty much because most other people did it, and also because it’s kind of nice. Especially for me working from home, I’m on a screen all day long. […] but then at the same time I felt like you don’t really get to connect with the other people*.

(Wurz et al. (2023) [52]|Facilitated Virtual Multidimensional Yoga Program)

For some participants, virtual support groups inhibited the ability to form connections. Some AYAs with cancer were members of other support groups. Others felt the provision of all necessary cancer-related information left little room for queries and discussions, resulting in reduced community interactions [35,41,42,45,52]. Some expressed in-person interventions facilitated better peer connections compared to the virtual mode. [41] A few AYAs desired support with mental health or two-way communication with HCPs [30,48].

#### 3.3.4. Autonomy

The positive contribution of the digital supportive care interventions in enhancing participants’ health, communication, and connection enabled them to make autonomous decisions about their bodies and health. Appropriate content and the “*freedom to set their behaviour goals and pace to meet their goals*” were important attributes for participants to gain a sense of agency [36,43,44,45,46,51]. The knowledge gained from cancer-specific modules gradually enabled participants to overcome the social stigma and embarrassment of seeking help for mental health [37,49]. AYAs felt more in control over their diagnosis, achieved a greater awareness of their symptoms, and learned new self-management skills. Inclusive and supportive peer systems enabled AYAs with cancer with the confidence to “*get through [cancer] and get well on the other side and get started in life again*” [35].

*Because of this group, I was able to let go of the emotional weight I was carrying with me, and finally, after 3 years I got my first haircut, and I feel like myself again. I feel like I am free to make my own decisions.*

(Lichiello et al. (2022) [36]|Facilitated Virtual Psychosocial Telehealth Intervention)

Positive psychology helped with feelings of competency, positive body image and a sense of autonomy among the AYAs with cancer [31,40,41,51,52]. The use of educational modules encouraged AYAs with cancer to explore new ways to overcome psychological challenges and build accountability. Specifically, for sexual health, these modules enabled AYAs to reduce stereotypes about sexuality [33] and empower couples to affirm the strength of their relationships through “teamwork”, reflections and open discussions [32,33].

## 4. Discussion

The results of this systematic review show that participants expressed having positive experiences with the digital supportive cancer care interventions, especially when tailored to the needs of AYAs with cancer. The identified interventions were found to benefit AYAs’ health and well-being, either as need-specific information [30,39,49], motivation for healthy routines [33,45,46,51], positive psychology skills (i.e., meditation and relaxation techniques) [31,33,36,39,41,44] or simply a source of enjoyment [32,44,48]. Most digital interventions contained a psychosocial element, indicating the importance of improving mental well-being for AYAs with cancer. Interventions designed to provide symptom management support (*n* = 2) were all delivered as software applications [47,48].

The majority of the interventions were designed to be asynchronous [30,31,32,33,35,37,39,40,42,44,46,47,48,49], containing videos, audio and session recordings. An asynchronous setting promotes individual autonomy through accessibility and the flexibility to set their own pace; this is vital to AYAs with cancer who may be in treatment or attending medical appointments. Despite being asynchronous, some interventions offered two-way communication such that participants were still able to interact and receive tailored content and personalized feedback from facilitators [32,33,37,45,46,49]. The majority of interventions with two-way communication were synchronous and offered real-time interactions with the facilitators and peers [34,36,38,41,43,50,51,52]. Although these sessions can be scheduled to accommodate AYAs’ school, work or medical appointments, AYAs expressed inconvenience with these interventions due to the limited choices on time and location [41].

There were instances where participants perceived certain aspects of the intervention attributes as barriers leading to discontinuation. Participants commonly withdrew from participating in the intervention because they were uninterested or preoccupied with the following factors: the intervention was available elsewhere, time or treatment-related conflicts, and the intervention felt too daunting [37,42,48,49,52]. Some AYAs with cancer were reluctant to fully commit as they wished to leave their cancer experiences behind and return to self-perceived normalcy [35,37,39,42,43]. It is important for researchers to explore the reasons for attrition in digital interventions to enhance understanding and better implementation of future updates and designs.

Only seven digital interventions in our review were designed pre-pandemic, dating back to 2014. The COVID-19 pandemic has undoubtedly acted as a catalyst in the adoption of digital healthcare and emphasized the need for virtual care, especially amidst the delays of in-person supportive care deliveries [53]. Some of the included interventions highlight the significant impact of the pandemic on cancer support services, research teams and AYAs with cancer [30,35,36,41,42,44]. The virtual format of peer support in some interventions encouraged participation [35,36,44]. While AYAs reported feeling validated by sharing with peers, they also expressed feeling a sense of loss to cancer and COVID-19. There were also disruptions in recruitment and operational constraints, resulting in early closures [30,42]. One psychosocial intervention, pivoting from in-person to virtual music therapy due to public health safety measures, had participants citing mixed experiences [41]. As participants were exposed to the features of the intervention in both modes, they indicated preferences for the previously offered in-person mode. This finding suggests that considering favourable aspects of in-person interventions in the design of digital supportive care interventions will enhance the participants’ overall experiences.

There may be inherent acceptability from AYAs with cancer for digital interventions due to their practicality and accessibility; however, limitations should also be acknowledged. The identified interventions did not appear to consider the racial and ethnic sensitivities of the participants. Fourteen studies included information on participants’ self-reported race and ethnicity as descriptive statistics [30,31,32,33,34,36,37,38,41,42,45,46,47,52]. However, no further sub-analyses based on race, ethnicity or other socioeconomic factors were explored. This limits our ability to draw conclusions regarding how some of these factors may have influenced participants’ experiences with the digital interventions. To enable broader adoption of digital supportive care interventions, an equity-based approach must be considered. Only one intervention in our review focused on individuals from rural communities [50,51]. Only one integrated virtual technology into an existing in-person support group intervention to improve attendance and reduce geographic disparities in access to care [38]. Additional empirical work is needed to explore the experiences of marginalized populations with digital interventions.

Most interventions assumed that the participants had the digital literacy, access to the internet and compatible devices required to participate in the intervention, with only five interventions explicitly providing the necessary technology (i.e., compatible devices, headphones, etc.) [36,38,45,46,48]. However, an over-reliance on the virtual nature to expand reach and access to underserved AYAs may create further inequity in supportive care delivery for cancer. This is particularly true among AYAs of lower socioeconomic status or those in regional or rural communities where certain barriers may be more prevalent, such as reduced access to a secure internet connection. Hence, further consideration is needed for participants who may not have the appropriate technology and digital literacy required to experience the intervention.

Most interventions included in the review were designed for all AYAs with cancer, irrespective of their type and stage. Current trends report an increase in AYAs being diagnosed with more advanced cancer stages [54,55,56]; however, only one intervention in this review considered the cancer stage of their population [32,33]. As such, more stage-specific supportive care interventions may be needed to ensure that appropriate content is being delivered to ensure patient-centred cancer care [57,58].

### 4.1. Study Limitations

This systematic review covers relatively narrow age and disease criteria, which means that while the included studies could provide valuable insights for AYAs with cancer, the findings may not be generalizable. Nine studies included in the review have a sample size of fewer than 15 participants, which further limits the robustness and applicability of the interventions to a larger population. There is also a risk that not all potential studies were included, given that a gray literature search was not conducted. Furthermore, the verbatim quotes used in the synthesis consist of data extracted from the published articles. This may have resulted in missing potentially richer insights, as authors may not have reported all relevant details. Finally, there were methodological limitations and minor concerns about adequacy and coherence as identified through the CASP checklist and GRADE-CERQual framework for the included studies. This may potentially affect the quality of the thematic findings.

### 4.2. Future Directions

The current definition of AYAs with cancer encompasses individuals aged 15–39 years. Our findings suggest that challenges faced by this population may extend beyond this age range. For instance, the experiences and needs of patients in their forties may align closer to AYAs than older patients, particularly in areas such as fertility concerns, career disruptions, and family responsibilities [30,32,33,39,49]. This age cut-off may inadvertently exclude patients who may benefit from AYA-specific supportive care interventions. The findings from this review indicate that a more flexible, needs-based approach to defining AYAs with cancer may be more appropriate, which could involve extending the upper age limit or focusing on the life stage rather than the chronological age. While redefining the age range alone may not capture the complexity of their needs and challenges, we believe it may be beneficial to consider and further explore the necessity of age- or cohort-specific interventions; especially in the cases of fertility concerns and psychosocial challenges resulting from cancer treatment. The aim is not to replace cancer-specific or need-specific interventions but rather complement them with a more nuanced approach by considering life-stage factors that go beyond age alone.

## 5. Conclusions

AYAs with cancer reported mostly favorable experiences with digital supportive care interventions. They appreciated supportive care interventions that are inclusive and accessible. Further analysis suggests that AYAs’ experiences with the embedded intervention attributes (e.g., choice, content, technology and environment) yield varied outcomes. Optimal outcomes included improvement in physical and psychological health, formation of connections, enhancement of communication and gain in autonomy.

## Figures and Tables

**Table 1:** Summary of identified intervention studies.

Study, Country	Delivery Mode	Intervention Description	Cancer Type and Cancer Phase	Age Range (m*)	AYA Specific	Discourse	Study Design	Sample Size	Data Collection and Analysis
**Psychosocial Supportive Care Interventions**									
Conduit et al. (2022) [30] Australia	Self-directed website	An information interface for testicular cancer with peer-to-peer support from a trained survivor to alleviate distress	Testicular and Diagnosis	24–55 (32.4)	No	2-way	Mixed methods	39	Interview and thematic analysis
Donovan et al. (2019) [31] USA	Multiple	A mobile-based delivery of mindfulness audio exercises and a Facebook-based peer support group	Sarcoma and Treatment and Survivorship	13–25 (19.3)	Yes	2-way	Qualitative	17	Interview and thematic analysis
Fergus et al. (2017) [32] Canada	Self-directed website	Understanding meanings from the creative expression exercise in the Fergus 2014 program to support couples’ coping	Breast and Multi-phase	18–40 (33.6)	No	1-way	Mixed methods	13	Open-ended survey questionnaire and content analysis
Fergus et al. (2014) [33] Canada	Self-directed website	Promotion of dyadic coping and adjustment through modules on intimacy, relationship awareness, constructive listening and communication skills	Breast and Multi-phase	<40 (34)	No	1-way	Mixed methods	10	Open-ended survey questionnaire, interviews and thematic analysis
Greer et al. (2019) [34] USA	Peer Support System	AI chatbot on Facebook to deliver positive psychology through chatting and survivor videos	Mixed and Survivorship	18–29 (25)	Yes	2-way	Mixed methods	45	Open-ended survey questionnaire, texts, forums and thematic analysis
Hanghøj et al. (2023) [35] Denmark	Software application	A social forum app to build a community for AYAs with cancer, provide information, and strengthen understanding and management of the illness and its symptoms.	Mixed and Diagnosis and Treatment	15–29 (24)	Yes	1-way	Mixed methods	75	Open-ended survey questionnaire, interviews and thematic analysis
Lichiello et al. (2022) [36] USA	Facilitated virtual program	8-week telehealth intervention with 60-min sessions derived from Acceptance and Commitment Therapy and Meaning-Centered Psychotherapy	Mixed and Multi-phase	18–39 (26)	Yes	1-way	Mixed methods	8	Open-ended survey questionnaire and thematic analysis
Markwardt et al. (2022) [37] USA	Facilitated virtual program	A personalized AYA care plan with evidence-based self-help interventions	Mixed, and Treatment and Survivorship	18–39 (31.64)	Yes	2-way	Qualitative	11	Interviews and thematic analysis
Melton et al. (2017) [38] USA	Facilitated virtual program	HIPAA-compliant videoconferencing application providing supportive group psychotherapy	Mixed and Multi-phase	18–40 (30.1)	Yes	2-way	Mixed methods	8	Open-ended survey questionnaire and thematic analysis
Micaux et al. (2020) [39] Sweden	Self-directed website	The psychoeducational intervention focuses on post-cancer sexual dysfunction and fertility distress	Mixed and Multi-phase	19–40 (33)	Yes	2-way	Qualitative	28	Interview and content analysis
Perumbil et al. (2022) [40] Australia	Self-directed website	A mindfulness-based interactive e-book with modules on paying attention five senses and self-care	Mixed and Treatment and Survivorship	14–29 (21.65)	Yes	1-way	Mixed methods	20	Open-ended survey questionnaire, interviews and thematic analysis
Phillips et al. (2023) [41] USA	Facilitated virtual program	Mindfulness-based music therapy intervention for anxiety and stress before and during the COVID-19 Pandemic	Mixed and Treatment	20–39 (31.5)	Yes	1-way	Qualitative	16	Interviews and content analysis
Poort et al. (2021) [42] USA	Software application	A combination of psychoeducational resources, coping skills training, and the opportunity to connect with peers	Mixed and Multi-phase	18–39 (28)	Yes	2-way	Qualitative	25	Interview and thematic analysis
Sansom-Daly et al. (2019) [43] Australia	Facilitated virtual program	Six group-based online sessions on cognitive-behavioral intervention consisting of psycho-educational information and exercises.	Mixed and Survivorship	15–25 (20.6)	Yes	2-way	Mixed methods	24	Open-ended survey questionnaire and content analysis
**Physical Activity (PA) Supportive Care Interventions**									
Barnes et al. (2023) [44] UK	Facilitated virtual program	8-week tailored exercise plan, with weekly calls to a cancer rehab specialist for tailored support	Mixed and Multi-phase	13–30 (22)	Yes	2-way	Mixed methods	57	Open-ended survey questionnaire and content analysis
Mendoza et al. (2017) [45] USA	Multiple	Activity-tracking wearable device paired with an app and a virtual peer support group on Facebook to promote PA.	Mixed and Survivorship	14–18 (16.6)	Yes	2-way	Mixed methods	22	Open-ended survey questionnaire, interviews and thematic analysis
Miropolsky et al. (2020) [46] USA	Multiple	Activity-tracking wearable device paired with an app and a virtual peer support group on Facebook peer support group with goal setting and a buddy system (adapted from Mendoza et al., 2020)	Mixed and Survivorship	20–39 (33.8)	Yes	2-way	Mixed methods	13	Interviews and thematic analysis
**Symptom Management Supportive Care Interventions**									
Erickson et al. (2018) [47] USA	Software Application	A heuristic tool to assist with symptom expression, illustrate their symptom experiences and enhance self-management	Mixed and Treatment	15–29 (20.9)	Yes	1-way	Mixed methods	83	Open-ended survey questionnaire, interviews and content analysis
Jibb et al. (2017) [48] Canada	Software Application	28-day Pain Squad+ pilot to provide real-time pain management	Mixed and Multi-phase	12–18	Yes	2-way	Qualitative	19	Interview and content analysis
**Multidimensional Supportive Care Interventions**									
Heiniger et al. (2017) [49] Australia	Multiple	An information hub on testicular cancer to reduce anxiety and depression	Testicular and Survivorship	27–57 (37.6)	No	1-way	Mixed methods	6	Interview and thematic analysis
Price & Brunet (2021) [50] Canada	Facilitated virtual program	Assessing the feasibility of a theory-based 12-week telehealth behaviour change intervention to promote PA and nutrition in rural areas	Mixed and Survivorship	20–39 (22.9)	Yes	2-way	Mixed methods	7	Interview and thematic analysis
Price & Brunet (2022) [51] Canada	Facilitated virtual program	Understanding the motivation of the participants in the 2021 telehealth behaviour change intervention as they adapt the PA and nutrition-based plan	Mixed and Survivorship	20–39 (33.9)	Yes	2-way	Mixed methods	7	Open-ended survey questionnaire, interviews and thematic analysis
Wurz et al. (2023) [52] Canada	Facilitated virtual program	An 8-week yoga intervention (60-min class per week) for AYAs with cancer and their support persons	Mixed and Multi-phase	18–39 (34.2)	Yes	1-way	Mixed methods	28	Open-ended survey questionnaire, interviews and content analysis

*m = mean or median age in years as specified in the studies.

## Data Availability

The datasets used and/or analyzed during the current study are available from the corresponding author upon reasonable request.

## Supplementary Materials

The following supporting information can be downloaded at https://www.mdpi.com/article/10.3390/cancers17050736/s1; Table S1: Search strategy for CINAHL; Table S2: Search strategy for PsycINFO; Table S3: Search strategy for EMBASE; Table S4: PRISMA 2020 main checklist; Table S5: Coding framework to capture the experiences of AYAs with cancer when using digital supportive care interventions; Table S6: CASP CHECKLIST; Table S7: GRADE-CERQual assessment of confidence.

## Abbreviations

The following abbreviations are used in this manuscript:

AYA	Adolescent and Young Adults
CASP	Critical Appraisal Skill Programme
COVID	Coronavirus disease
HCP	Healthcare Provider
PRISMA	Preferred Reporting Items for Systematic Reviews and Meta-Analyses
GRADE-CERQual	Grading of Recommendations Assessment, Development and Evaluation- Confidence in Evidence from Reviews of Qualitative research

## Appendix A

**Table A1:** Search strategy for MEDLINE (Ovid).

Records before removing duplicates: 1874 Original searches (exactly as executed):	
**#1**	Adolescent/
**#2**	adolescent*.ti,ab,hw,kf.
**#3**	(teen* or youth* or AYA).ti,ab,hw,kf.
**#4**	Young Adult/
**#5**	((young or emerging) adj2 (adult* or person* or individual* or people* or population* or man or men or wom#n)).ti,ab,hw,kf.
**#6**	((highschool* or college* or university or “secondary school”) adj2 student*).ti,ab,hw,kf.
**#7**	Adult/
**#8**	adult*.ti,ab,hw,kf.
**#9**	or/1–8
**#10**	exp Neoplasms/
**#11**	Cancer Survivors/
**#12**	(neoplasm* or cancer* or oncolog* or malignan* or tumor* or tumour*).ti,ab,hw,kf.
**#13**	or/10–12
**#14**	Videoconferencing/
**#15**	telemedicine/or remote consultation/or telerehabilitation/
**#16**	(e-health or ehealth or m-health or telehealth or telemedicine).ti,ab,hw,kf.
**#17**	((virtual or remote or digital or mobile or online or hybrid) adj3 (care or health* or intervention*)).ti,ab,hw,kf.
**#18**	((electronic or mobile or digital) adj device*).ti,ab,hw,kf.
**#19**	social networking/or online social networking/
**#20**	Attitude to Computers/
**#21**	exp Internet/
**#22**	Internet-Based Intervention/
**#23**	Mobile Applications/
**#24**	cell phone/or smartphone/or text messaging/
**#25**	(app or apps or smartphone* or “social media” or internet or “text message*” or web-based).ti,ab,hw,kf.
**#26**	(facebook or zoom or fitbit or instagram).ti,ab,hw,kf.
**#27**	exp Monitoring, Physiologic/
**#28**	Fitness Trackers/
**#29**	(activity adj1 (monitor* or tracker*)).ti,ab,hw,kf.
**#30**	or/14–29
**#31**	exp Exercise/
**#32**	Exercise Therapy/
**#33**	exp Nutrition Therapy/
**#34**	Diet/
**#35**	Mental Health/
**#36**	Psychological distress/or Psycho-oncology/or Psychotherapy/
**#37**	exp “Quality of Life”/
**#38**	Counseling/
**#39**	psychosocial intervention/
**#40**	Mind–Body therapies/
**#41**	Spiritual therapies/or Pastoral care/
**#42**	Social support/
**#43**	Sexual Health/
**#44**	Pain Management/
**#45**	Palliative care/
**#46**	exp rehabilitation/
**#47**	(exercise* or nutrition or diet or “physical activit*” or “mental health” or counsel?ing or psychotherap* or palliative or “physical therap*” or physiotherap* or “mind–body” or “sexual health”).ti,ab,hw,kf.
**#48**	(spiritual adj1 (care or therap*)).ti,ab,hw,kf.
**#49**	(pastoral adj1 (care or therap*)).ti,ab,hw,kf.
**#50**	(pain adj2 manage*).ti,ab,hw,kf.
**#51**	(supportive adj1 care).ti,ab,hw,kf.
**#52**	sleep/or fatigue/or “Sleep Initiation and Maintenance Disorders”/
**#53**	anxiety/or depression/
**#54**	(anxiety* or depress*).ti,ab,hw,kf.
**#55**	(sleep* or fatigue or insomnia or anxiety or depress*).ti,ab,hw,kf.
**#56**	or/31–55
**#57**	exp qualitative research/
**#58**	Grounded theory/
**#59**	qualitative.ti,ab,hw,kf.
**#60**	(interview* or “focus group*” or diary or open-ended or narrative).ti,ab,hw,kf.
**#61**	((face or f2f or “face-to-face” or guide* or depth or indepth or “in-depth” or informal or semistructured or “semi-structured” or structured or unstructured) adj3 (discussion* or interview* or questionnaire*)).ti,ab,hw,kf.
**#62**	(ethnograph* or (field adj1 work) or fieldwork or (focus adj1 (group or groups)) or (key adj1 informant*) or qualitative).ti,ab,hw,kf.
**#63**	(“grounded theory” or “content analysis” or “framework analysis” or “thematic analysis”).ti,ab,hw,kf.
**#64**	(experience* or impression* or evaluat*).ti,ab,hw,kf.
**#65**	interview/
**#66**	interviews as topic/
**#67**	Focus Groups/
**#68**	narration/
**#69**	or/57–68
**#70**	The Pediatric Cancer Survivors’ User Experiences With Digital Health Interventions.m_titl.
**#71**	“Young adult cancer survivors’ experience of taking part in a 12-week exercise referral programme: a qualitative study of the Trekstock RENEW initiative.”.m_titl.
**#72**	(Participant Perceptions on a Fitbit and Facebook Intervention for Young Adult Cancer Survivors: A Qualitative Study).m_titl.
**#73**	70 or 71 or 72
**#74**	9 and 13 and 30 and 56 and 69
**#75**	74 or 73
**#76**	75 not 74
**#77**	limit 74 to (english language and yr = “2000 –Current”)

## References

[1] GBD 2019 Adolescent Young Adult Cancer Collaborators (2022). The global burden of adolescent and young adult cancer in 2019: A systematic analysis for the Global Burden of Disease Study 2019. Lancet Oncol..

[2] (2015). Adolescents and Young Adults (AYAs) with Cancer—NCI. https://www.cancer.gov/types/aya.

[3] Avery J., Wong E., Harris C., Chapman S., Uppal S., Shanawaz S., Edwards A., Burnett L., Vora T., Gupta A.A. (2022). The Transformation of Adolescent and Young Adult Oncological and Supportive Care in Canada: A Mixed Methods Study. Curr. Oncol..

[4] Trama A., Botta L., Foschi R., Ferrari A., Stiller C., Desandes E., Maule M.M., Merletti F., Gatta G., EUROCARE-5 Working Group (2016). Survival of European adolescents and young adults diagnosed with cancer in 2000–2007: Population-based data from EUROCARE-5. Lancet Oncol..

[5] Ferrari A., Stark D., Peccatori F.A., Fern L., Laurence V., Gaspar N., Bozovic-Spasojevic I., Smith O., De Munter J., Derwich K. (2021). Adolescents and young adults (AYA) with cancer: A position paper from the AYA Working Group of the European Society for Medical Oncology (ESMO) and the European Society for Paediatric Oncology (SIOPE). ESMO Open.

[6] Itzep N., Roth M. (2022). Psychosocial Distress Due to Interference of Normal Developmental Milestones in AYAs with Cancer. Children.

[7] Ben-Ari E. (2021). New Task Force Focuses on Quality of Life for AYAs with Cancer. National Cancer Institute. https://www.cancer.gov/news-events/cancer-currents-blog/2021/aya-cancer-patient-reported-quality-of-life.

[8] Physical Activity and the Person with Cancer. https://www.cancer.org/cancer/survivorship/be-healthy-after-treatment/physical-activity-and-the-cancer-patient.html.

[9] Redmond K. (1996). Advances in supportive care. Eur. J. Cancer Care.

[10] Scotté F., Taylor A., Davies A. (2023). Supportive Care: The “Keystone” of Modern Oncology Practice. Cancers.

[11] Kangas M., Henry J.L., Bryant R.A. (2002). Posttraumatic stress disorder following cancer: A conceptual and empirical review. Clin. Psychol. Rev..

[12] Kissane D.W., Grabsch B., Clarke D.M., Smith G.C., Love A.W., Bloch S., Snyder R.D., Li Y. (2007). Supportive-expressive group therapy for women with metastatic breast cancer: Survival and psychosocial outcome from a randomized controlled trial. Psychooncology.

[13] British Columbia Ministry of Health (2021). How Virtual Care Is Meeting the Needs of Patients in the COVID-19 Landscape and Beyond: Patient Stories. A Picture of Health—Ministry of Health Newsletter. https://www2.gov.bc.ca/assets/gov/health/about-bc-s-health-care-system/heath-care-partners/health-newsletter/patient-stories-virtual-care-2021.pdf.

[14] Barriers and Strategies to Equity in Digital Health Programs and Services. https://www.publichealthontario.ca/-/media/Documents/E/2023/eb-barriers-strategies-equity-digital-health-programs-services.pdf.

[15] Hatef E., Wilson R.F., Hannum S.M., Zhang A., Kharrazi H., Weiner J.P., Davis S.A., Robinson K.A. (2023). Use of Telehealth During the COVID-19 Era.

[16] Vogels E.A. (2019). Millennials Stand out for Their Technology Use, But Older Generations Also Embrace Digital Life. Pew Research Center. https://www.pewresearch.org/short-reads/2019/09/09/us-generations-technology-use/.

[17] Ridout B., Campbell A. (2018). The Use of Social Networking Sites in Mental Health Interventions for Young People: Systematic Review. J. Med. Internet Res..

[18] Lazard A.J., Saffer A.J., Horrell L., Benedict C., Love B. (2020). Peer-to-peer connections: Perceptions of a social support app designed for young adults with cancer. Psychooncology.

[19] Williamson Lewis R., Howell K.E., Effinger K.E., Meacham L.R., Wasilewski-Masker K., Mertens A., Gilleland Marchak J. (2023). Feasibility of Fitbit Use in Adolescent Survivors of Pediatric Cancer: Who Consistently Uses It and for How Long?. J. Adolesc. Young Adult Oncol..

[20] Wanberg L.J., Kim A., Vogel R.I., Sadak K.T., Teoh D. (2023). Usability and Satisfaction Testing of Game-Based Learning Avatar-Navigated Mobile (GLAm), an App for Cervical Cancer Screening: Mixed Methods Study. JMIR Form. Res..

[21] Kock A.-K., Kaya R.S., Müller C., Andersen B., Langer T., Ingenerf J. (2015). Design, Implementation, and Evaluation of a Mobile Application for Patient Empowerment and Management of Long-Term Follow-Up after Childhood Cancer. Klin. Pädiatr..

[22] Cheng L., Liu F., Mao X., Peng W., Wang Y., Huang H., Duan M., Wang Y., Yuan C. (2022). The Pediatric Cancer Survivors’ User Experiences with Digital Health Interventions: A Systematic Review of Qualitative Data. Cancer Nurs..

[23] RAYYAN. https://www.rayyan.ai/.

[24] Page M.J., McKenzie J.E., Bossuyt P.M., Boutron I., Hoffmann T.C., Mulrow C.D., Shamseer L., Tetzlaff J.M., Akl E.A., Brennan S.E. (2021). The PRISMA 2020 statement: An updated guideline for reporting systematic reviews. BMJ.

[25] (2023). Lumivero. NVivo. www.lumivero.com.

[26] Thomas J., Harden A. (2008). Methods for the thematic synthesis of qualitative research in systematic reviews. BMC Med. Res. Methodol..

[27] Critical Appraisal Skills Programme (2023). CASP Qualitative Research Checklist. https://casp-uk.net/casp-tools-checklists/.

[28] (2024). GRADE-CERQual_Home. https://www.cerqual.org/.

[29] Haddaway N.R., Page M.J., Pritchard C.C., McGuinness L.A. (2022). PRISMA2020: An R package and Shiny app for producing PRISMA 2020-compliant flow diagrams, with interactivity for optimised digital transparency and Open Synthesis. Campbell Syst. Rev..

[30] Conduit C., Guo C., Smith A.B., Rincones O., Baenziger O., Thomas B., Goad J., Lenaghan D., Lawrentschuk N., Wong L.-M. (2022). Role for a Web-Based Intervention to Alleviate Distress in People with Newly Diagnosed Testicular Cancer: Mixed Methods Study. JMIR Cancer.

[31] Donovan E., Martin S.R., Seidman L.C., Zeltzer L.K., Cousineau T.M., Payne L.A., Trant M., Weiman M., Knoll M., Federman N.C. (2019). A Mobile-Based Mindfulness and Social Support Program for Adolescents and Young Adults with Sarcoma: Development and Pilot Testing. JMIR mHealth uHealth.

[32] Fergus K., Ahmad S., Ianakieva I., McLeod D., Carter W. (2017). Metaphor and meaning in an online creative expression exercise to promote dyadic coping in young couples affected by breast cancer. Arts Health Int. J. Res. Policy Pract..

[33] Fergus K.D., Mcleod D., Carter W., Warner E., Gardner S.L., Cullen K.I. (2014). Development and pilot testing of an online intervention to support young couples’ coping and adjustment to breast cancer. Eur. J. Cancer Care.

[34] Greer S., Ramo D., Chang Y.-J., Fu M., Moskowitz J., Haritatos J. (2019). Use of the Chatbot “Vivibot” to Deliver Positive Psychology Skills and Promote Well-Being Among Young People After Cancer Treatment: Randomized Controlled Feasibility Trial. JMIR mHealth uHealth.

[35] Hanghøj S., Bentsen L., Hjerming M., Simonsen A.B., Thycosen M., Bergmann M.B., Godiksen D.Ø., Pappot H. (2023). Experiences of Peer Communities in a Cancer Smartphone App Among Adolescents and Young Adults with Cancer. Semin. Oncol. Nurs..

[36] Lichiello S., Rainwater L., Russell G.B., Pulgar C., Clark J., Daniel S., McCall M.H., Bentley P., Duckworth K.E. (2022). Cancer during a pandemic: A psychosocial telehealth intervention for young adults. Curr. Probl. Cancer.

[37] Markwardt H.S., Taghavi S.E., Williams A.P., Olivares M.N., McDuffee P.R., Al Achkar M., Hall B.C. (2022). The AYA Care Plan: Initial Evaluation of a Web-Based Psychosocial Intervention. JCO Clin. Cancer Inform..

[38] Melton L., Brewer B., Kolva E., Joshi T., Bunch M. (2017). Increasing access to care for young adults with cancer: Results of a quality-improvement project using a novel telemedicine approach to supportive group psychotherapy. Palliat. Support. Care.

[39] Micaux Obol C., Lampic C., Wettergren L., Ljungman L., Eriksson L.E. (2020). Experiences of a web-based psycho-educational intervention targeting sexual dysfunction and fertility distress in young adults with cancer-A self-determination theory perspective. PLoS ONE.

[40] Perumbil Pathrose S., Patterson P., Ussher J., Everett B., Salamonson Y., McDonald F., Biegel G.M., He S., Ramjan L. (2022). Feasibility, Acceptability, and Psychosocial Outcomes of a Mindfulness-Based Interactive e-Book for Young People with Cancer. J. Adolesc. Young Adult Oncol..

[41] Phillips C.S., Bockhoff J., Berry D.L., Buchbinder E., Frazier A.L., LaCasce A., Ligibel J., Luskin M.R., Woods H., Knoerl R. (2023). Exploring Young Adults’ Perspectives of Participation in a Mindfulness-Based Music Therapy Intervention Before and During the COVID-19 Pandemic. J. Adolesc. Young Adult Oncol..

[42] Poort H., Ryan A., MacDougall K., Malinowski P., MacDonald A., Markin Z., Pirl W., Greer J., Fasciano K. (2021). Feasibility and Acceptability of a Mobile Phone App Intervention for Coping with Cancer as a Young Adult: Pilot Trial and Thematic Analysis. J. Med. Internet Res..

[43] Sansom-Daly U.M., Wakefield C.E., Bryant R.A., Patterson P., Anazodo A., Butow P., Sawyer S.M., McGill B.C., Evans H.E., Cohn R.J. (2019). Feasibility, acceptability, and safety of the Recapture Life videoconferencing intervention for adolescent and young adult cancer survivors. Psychooncology.

[44] Barnes E., Hillier-Moses G., Murray H., Stevinson C., Franks H.A., Gossage L. (2023). Evaluation of the MOVE online exercise programme for young people aged 13–30. Support. Care Cancer.

[45] Mendoza J.A., Baker K.S., Moreno M.A., Whitlock K., Abbey-Lambertz M., Waite A., Colburn T., Chow E.J. (2017). A Fitbit and Facebook mHealth intervention for promoting physical activity among adolescent and young adult childhood cancer survivors: A pilot study. Pediatr. Blood Cancer.

[46] Miropolsky E.M., Scott Baker K., Abbey-Lambertz M., Syrjala K., Chow E.J., Ceballos R., Mendoza J.A. (2020). Participant Perceptions on a Fitbit and Facebook Intervention for Young Adult Cancer Survivors: A Qualitative Study. J. Adolesc. Young Adult Oncol..

[47] Erickson J.M., Ameringer S., Linder L., Macpherson C.F., Elswick R.K.J., Luebke J.M., Stegenga K. (2019). Using a Heuristic App to Improve Symptom Self-Management in Adolescents and Young Adults with Cancer. J. Adolesc. Young Adult Oncol..

[48] Jibb L.A., Cafazzo J.A., Nathan P.C., Seto E., Stevens B.J., Nguyen C., Stinson J.N. (2017). Development of a mHealth Real-Time Pain Self-Management App for Adolescents with Cancer: An Iterative Usability Testing Study. J. Pediatr. Oncol. Nurs..

[49] Heiniger L.E., Smith A.B., Olver I., Grimison P., Klein B., Wootten A., Abbott J.-A.m., Price M.a., McJannett M., Tran B. (2017). e-TC: Development and pilot testing of a web-based intervention to reduce anxiety and depression in survivors of testicular cancer. Eur. J. Cancer Care.

[50] Price J., Brunet J. (2021). Feasibility and acceptability of a telehealth behavior change intervention for promoting physical activity and fruit and vegetable consumption among rural-living young adult cancer survivors. J. Psychosoc. Oncol..

[51] Price J., Brunet J. (2022). Understanding rural-living young adult cancer survivors’ motivation during a telehealth behavior change intervention within a single-arm feasibility trial. Health Inform. J..

[52] Wurz A., McLaughlin E., Hughes K., Ellis K., Chen A., Cowley L., Molina H., Duchek D., Eisele M., Culos-Reed S.N. (2023). Exploring feasibility, perceptions of acceptability, and potential benefits of an 8-week yoga intervention delivered by videoconference for young adults affected by cancer: A single-arm hybrid effectiveness-implementation pilot study. Pilot Feasibility Stud..

[53] Keesara S., Jonas A., Schulman K. (2020). Covid-19 and Health Care’s Digital Revolution. N. Engl. J. Med..

[54] Winestone L.E., Wilkes J.J., Puccetti D., Keegan T.H.M., Henk H.J., McPheeters J., Kahn J.M., Ginsberg J., Wong S., Timberline S. (2024). Time to diagnosis among young patients with cancer. Pediatr. Blood Cancer.

[55] Janardan S.K., Wechsler D.S. (2021). Caught in the In-Between: Challenges in Treating Adolescents and Young Adults with Cancer. JCO Oncol. Pract..

[56] Larsen L.L.V. (2023). “Could this be cancer?” Addressing diagnostic delay disparities in adolescents and young adults with cancer through targeted awareness and mitigation of barriers to help-seeking behaviors. J. Psychosoc. Oncol. Res. Pract..

[57] Balogh E.P., Ganz P.A., Murphy S.B., Nass S.J., Ferrell B.R., Stovall E. (2011). Patient-Centered Cancer Treatment Planning: Improving the Quality of Oncology Care. Summary of an Institute of Medicine Workshop. Oncologist.

[58] Elkefi S., Asan O. (2023). The Impact of Patient-Centered Care on Cancer Patients’ QOC, Self-Efficacy, and Trust Towards Doctors: Analysis of a National Survey. J. Patient Exp..

