# Supplementary material for: The Experiences of Adolescents and Young Adults with Digital Supportive Care Interventions for Cancer: A Systematic Review of Qualitative Studies
Source: Cancers (Basel). 2025 Feb 21;17(5):736. doi: 10.3390/cancers17050736 (PMC11899503; doi:10.3390/cancers17050736)
Supplement: Supplementary file 1 [file cancers-17-00736-s001.zip › Supplementary Table S1 (SS_CINAHL).pdf]

#	Query	Limiters/Expanders	Last Run Via	Results
S61	S6 AND S9 AND S27 AND S49 AND S58	Limiters - Publication Date: 20000101-20231231<br>Search modes - Proximity	Interface - EBSCOhost Research Databases<br>Search Screen - Advanced Search<br>Database - CINAHL Complete	Display
S60	S6 AND S9 AND S27 AND S49 AND S58	Limiters - Publication Date: 20000101-20231231<br>Search modes - Proximity	Interface - EBSCOhost Research Databases<br>Search Screen - Advanced Search<br>Database - CINAHL Complete	Display
S59	S6 AND S9 AND S27 AND S49 AND S58	Search modes - Proximity	Interface - EBSCOhost Research Databases<br>Search Screen - Advanced Search<br>Database - CINAHL Complete	Display
S58	S50 OR S51 OR S52 OR S53 OR S54 OR S55 OR S56 OR S57	Search modes - Proximity	Interface - EBSCOhost Research Databases<br>Search Screen - Advanced Search<br>Database - CINAHL Complete	Display
S57	(MH "Interviews+") OR (MH "Narratives") OR (MH "Self Report+")	Search modes - Proximity	Interface - EBSCOhost Research Databases<br>Search Screen - Advanced Search<br>Database - CINAHL Complete	Display
S56	TI (experience* OR impression* OR evaluat*) OR AB (experience* OR impression* OR evaluat*) OR SU (experience* OR impression* OR evaluat*)	Search modes - Proximity	Interface - EBSCOhost Research Databases<br>Search Screen - Advanced Search<br>Database - CINAHL Complete	Display
S55	TI ("grounded theory" OR "content analysis" OR	Search modes - Proximity	Interface - EBSCOhost Research Databases	Display

	"framework analysis" OR "thematic analysis") OR AB ("grounded theory" OR "content analysis" OR "framework analysis" OR "thematic analysis") OR SU ("grounded theory" OR "content analysis" OR "framework analysis" OR "thematic analysis")		Search Screen - Advanced Search<br>Database - CINAHL Complete	
S54	TI (ethnograph* OR (field N1 work) OR fieldwork OR (focus N1 (group OR groups)) OR (key N1 informant*)) OR AB (ethnograph* OR (field N1 work) OR fieldwork OR (focus N1 (group OR groups)) OR (key N1 informant*)) OR SU (ethnograph* OR (field N1 work) OR fieldwork OR (focus N1 (group OR groups)) OR (key N1 informant*))	Search modes - Proximity	Interface - EBSCOhost Research Databases<br>Search Screen - Advanced Search<br>Database - CINAHL Complete	Display
S53	TI ((face OR f2f OR "face-to-face" OR guide* OR depth OR indepth OR "in-depth" OR informal OR semistructured OR "semi-structured" OR structured OR unstructured) N3 (discussion* OR interview* OR questionnaire*)) OR AB ((face OR f2f OR "face-to-face" OR guide* OR depth OR indepth OR "in-depth" OR informal OR semistructured OR "semi-structured" OR structured OR unstructured) N3 (discussion* OR interview* OR questionnaire*)) OR	Search modes - Proximity	Interface - EBSCOhost Research Databases<br>Search Screen - Advanced Search<br>Database - CINAHL Complete	Display

	SU ((face OR f2f OR "face-to-face" OR guide* OR depth OR indepth OR "in-depth" OR informal OR semistructured OR "semi-structured" OR structured OR unstructured) N3 (discussion* OR interview* OR questionnaire*))			
S52	TI (interview* OR focus group* OR diary OR open-ended OR narrative) OR AB (interview* OR focus group* OR diary OR open-ended OR narrative) OR SU (interview* OR focus group* OR diary OR open-ended OR narrative)	Search modes - Proximity	Interface - EBSCOhost Research Databases<br>Search Screen - Advanced Search<br>Database - CINAHL Complete	Display
S51	TI qualitative OR AB qualitative OR SU qualitative	Search modes - Proximity	Interface - EBSCOhost Research Databases<br>Search Screen - Advanced Search<br>Database - CINAHL Complete	Display
S50	(MH "Qualitative Studies+")	Search modes - Proximity	Interface - EBSCOhost Research Databases<br>Search Screen - Advanced Search<br>Database - CINAHL Complete	Display
S49	S28 OR S29 OR S30 OR S31 OR S32 OR S33 OR S34 OR S35 OR S36 OR S37 OR S38 OR S39 OR S40 OR S41 OR S42 OR S43 OR S44 OR S45 OR S46 OR S47 OR S48	Search modes - Proximity	Interface - EBSCOhost Research Databases<br>Search Screen - Advanced Search<br>Database - CINAHL Complete	Display
S48	TI (sleep* OR fatigue OR insomnia) OR AB (sleep* OR fatigue OR insomnia) OR SU (sleep* OR fatigue OR insomnia)	Search modes - Proximity	Interface - EBSCOhost Research Databases<br>Search Screen - Advanced Search<br>Database - CINAHL Complete	Display

S47	(MH "Cancer Fatigue") OR (MH "Fatigue")	Search modes - Proximity	Interface - EBSCOhost Research Databases<br>Search Screen - Advanced Search<br>Database - CINAHL Complete	Display
S46	(MH "Insomnia")	Search modes - Proximity	Interface - EBSCOhost Research Databases<br>Search Screen - Advanced Search<br>Database - CINAHL Complete	Display
S45	(MH "Sleep+")	Search modes - Proximity	Interface - EBSCOhost Research Databases<br>Search Screen - Advanced Search<br>Database - CINAHL Complete	Display
S44	TI supportive N1 care OR AB supportive N1 care OR SU supportive N1 care	Search modes - Proximity	Interface - EBSCOhost Research Databases<br>Search Screen - Advanced Search<br>Database - CINAHL Complete	Display
S43	TI pain N2 manage* OR AB pain N2 manage* OR SU pain N2 manage*	Search modes - Proximity	Interface - EBSCOhost Research Databases<br>Search Screen - Advanced Search<br>Database - CINAHL Complete	Display
S42	TI ((spiritual OR pastoral) N1 (care OR therap*)) OR AB ((spiritual OR pastoral) N1 (care OR therap*)) OR SU ((spiritual OR pastoral) N1 (care OR therap*))	Search modes - Proximity	Interface - EBSCOhost Research Databases<br>Search Screen - Advanced Search<br>Database - CINAHL Complete	Display
S41	TI (exercise* OR nutrition OR diet OR physical activit* OR "mental health" OR counsel?ing OR psychotherap* OR palliative OR physical therap* OR physiotherap* OR "mind-body" OR	Search modes - Proximity	Interface - EBSCOhost Research Databases<br>Search Screen - Advanced Search<br>Database - CINAHL Complete	Display

	"sexual health" OR anxiety OR depress*) OR AB (exercise* OR nutrition OR diet OR physical activit* OR "mental health" OR counsel?ing OR psychotherap* OR palliative OR physical therap* OR physiotherap* OR "mind-body" OR "sexual health" OR anxiety OR depress*) OR SU (exercise* OR nutrition OR diet OR physical activit* OR "mental health" OR counsel?ing OR psychotherap* OR palliative OR physical therap* OR physiotherap* OR "mind-body" OR "sexual health" OR anxiety OR depress*)			
S40	(MH "Pain Management")	Search modes - Proximity	Interface - EBSCOhost Research Databases<br>Search Screen - Advanced Search<br>Database - CINAHL Complete	Display
S39	(MH "Sexual Health")	Search modes - Proximity	Interface - EBSCOhost Research Databases<br>Search Screen - Advanced Search<br>Database - CINAHL Complete	Display
S38	(MH "Support, Social") OR (MH "Support, Psychosocial")	Search modes - Proximity	Interface - EBSCOhost Research Databases<br>Search Screen - Advanced Search<br>Database - CINAHL Complete	Display
S37	(MH "Spiritual Care") OR (MH "Palliative Care")	Search modes - Proximity	Interface - EBSCOhost Research Databases	Display

			Search Screen - Advanced Search<br>Database - CINAHL Complete	
S36	(MH "Mind Body Techniques+")	Search modes - Proximity	Interface - EBSCOhost Research Databases<br>Search Screen - Advanced Search<br>Database - CINAHL Complete	Display
S35	(MH "Quality of Life") OR (MH "Psychological Well-Being")	Search modes - Proximity	Interface - EBSCOhost Research Databases<br>Search Screen - Advanced Search<br>Database - CINAHL Complete	Display
S34	(MH "Counseling+")	Search modes - Proximity	Interface - EBSCOhost Research Databases<br>Search Screen - Advanced Search<br>Database - CINAHL Complete	Display
S33	(MH "Psycho-Oncology") OR (MH "Rehabilitation, Cancer") OR (MH "Psychology, Clinical")	Search modes - Proximity	Interface - EBSCOhost Research Databases<br>Search Screen - Advanced Search<br>Database - CINAHL Complete	Display
S32	(MH "Psychological Distress") OR (MH "Anxiety") OR (MH "Depression")	Search modes - Proximity	Interface - EBSCOhost Research Databases<br>Search Screen - Advanced Search<br>Database - CINAHL Complete	Display
S31	(MH "Mental Health") OR (MH "Counseling") OR (MH "Psychotherapy+") OR (MH "Cognitive Therapy")	Search modes - Proximity	Interface - EBSCOhost Research Databases<br>Search Screen - Advanced Search<br>Database - CINAHL Complete	Display
S30	(MH "Diet Therapy")	Search modes - Proximity	Interface - EBSCOhost Research Databases<br>Search Screen - Advanced Search<br>Database - CINAHL Complete	Display

S29	(MH "Nutrition") OR (MH "Adolescent Nutrition") OR (MH "Diet")	Search modes - Proximity	Interface - EBSCOhost Research Databases<br>Search Screen - Advanced Search<br>Database - CINAHL Complete	Display
S28	(MH "Exercise+") OR (MH "Therapeutic Exercise+") OR (MH "Rehabilitation") OR (MH "Physical Therapy")	Search modes - Proximity	Interface - EBSCOhost Research Databases<br>Search Screen - Advanced Search<br>Database - CINAHL Complete	Display
S27	S10 OR S11 OR S12 OR S13 OR S14 OR S15 OR S16 OR S17 OR S18 OR S19 OR S20 OR S21 OR S22 OR S23 OR S24 OR S25 OR S26	Search modes - Proximity	Interface - EBSCOhost Research Databases<br>Search Screen - Advanced Search<br>Database - CINAHL Complete	Display
S26	TI (activity N1 (monitor* or tracker*)) OR AB (activity N1 (monitor* or tracker*)) OR SU (activity N1 (monitor* or tracker*))	Search modes - Proximity	Interface - EBSCOhost Research Databases<br>Search Screen - Advanced Search<br>Database - CINAHL Complete	Display
S25	TI (facebook or zoom or fitbit or instagram) OR AB (facebook or zoom or fitbit or instagram) OR SU (facebook or zoom or fitbit or instagram)	Search modes - Proximity	Interface - EBSCOhost Research Databases<br>Search Screen - Advanced Search<br>Database - CINAHL Complete	Display
S24	(MH "Fitness Trackers")	Search modes - Proximity	Interface - EBSCOhost Research Databases<br>Search Screen - Advanced Search<br>Database - CINAHL Complete	Display
S23	(MH "Monitoring, Physiologic")	Search modes - Proximity	Interface - EBSCOhost Research Databases<br>Search Screen - Advanced Search<br>Database - CINAHL Complete	Display
S22	TI (app or apps or smartphone* or "social media" or internet or text	Search modes - Proximity	Interface - EBSCOhost Research Databases<br>Search Screen - Advanced	Display

	message* or web-based OR video game* or "augmented reality" OR "virtual reality") OR AB (app or apps or smartphone* or "social media" or internet or text message* or web-based OR video game* or "augmented reality" OR "virtual reality") OR SU (app or apps or smartphone* or "social media" or internet or text message* or web-based OR video game* or "augmented reality" OR "virtual reality")		Search<br>Database - CINAHL Complete	
S21	(MH "Attitude to Computers") OR (MH "Computers, Portable+")	Search modes - Proximity	Interface - EBSCOhost Research Databases<br>Search Screen - Advanced Search<br>Database - CINAHL Complete	Display
S20	(MH "Mobile Applications")	Search modes - Proximity	Interface - EBSCOhost Research Databases<br>Search Screen - Advanced Search<br>Database - CINAHL Complete	Display
S19	(MH "Text Messaging") OR (MH "Instant Messaging") OR (MH "Internet-Based Intervention")	Search modes - Proximity	Interface - EBSCOhost Research Databases<br>Search Screen - Advanced Search<br>Database - CINAHL Complete	Display
S18	(MH "Online Social Networking")	Search modes - Proximity	Interface - EBSCOhost Research Databases<br>Search Screen - Advanced Search<br>Database - CINAHL Complete	Display
S17	(MH "Social Media") OR (MH "Internet") OR (MH	Search modes - Proximity	Interface - EBSCOhost Research Databases<br>Search Screen - Advanced	Display

	"Facebook") OR (MH "Twitter") OR (MH "Email")		Search<br>Database - CINAHL Complete	
S16	(MH "Cellular Phone") OR (MH "Smartphone")	Search modes - Proximity	Interface - EBSCOhost Research Databases<br>Search Screen - Advanced Search<br>Database - CINAHL Complete	Display
S15	TI ((electronic or mobile or digital) N1 device*)) OR AB ((electronic or mobile or digital) N1 device*)) OR SU ((electronic or mobile or digital) N1 device*))	Search modes - Proximity	Interface - EBSCOhost Research Databases<br>Search Screen - Advanced Search<br>Database - CINAHL Complete	Display
S14	TI ((virtual or remote or digital or mobile or online or hybrid) N3 (care or health* or intervention*)) OR AB ((virtual or remote or digital or mobile or online or hybrid) N3 (care or health* or intervention*)) OR SU ((virtual or remote or digital or mobile or online or hybrid) N3 (care or health* or intervention*))	Search modes - Proximity	Interface - EBSCOhost Research Databases<br>Search Screen - Advanced Search<br>Database - CINAHL Complete	Display
S13	TI (e-health or ehealth or m-health or telehealth or telemedicine) OR AB (e-health or ehealth or m-health or telehealth or telemedicine) OR SU (e-health or ehealth or m-health or telehealth or telemedicine)	Search modes - Proximity	Interface - EBSCOhost Research Databases<br>Search Screen - Advanced Search<br>Database - CINAHL Complete	Display
S12	(MH "Telemedicine") OR (MH "Telerehabilitation") OR (MH "Telepsychiatry") OR (MH "Telehealth") OR (MH "Telenursing") OR	Search modes - Proximity	Interface - EBSCOhost Research Databases<br>Search Screen - Advanced Search<br>Database - CINAHL Complete	Display

	(MH "Telenutrition") OR (MH "Remote Consultation")			
S11	(MH "Video Games") OR (MH "Virtual Reality") OR (MH "Augmented Reality")	Search modes - Proximity	Interface - EBSCOhost Research Databases<br>Search Screen - Advanced Search<br>Database - CINAHL Complete	Display
S10	(MH "Teleconferencing") OR (MH "Videoconferencing+")	Search modes - Proximity	Interface - EBSCOhost Research Databases<br>Search Screen - Advanced Search<br>Database - CINAHL Complete	Display
S9	S7 OR S8	Search modes - Proximity	Interface - EBSCOhost Research Databases<br>Search Screen - Advanced Search<br>Database - CINAHL Complete	Display
S8	TI (neoplasm* or cancer* or oncolog* or malignan* or tumor* or tumour*) OR AB (neoplasm* or cancer* or oncolog* or malignan* or tumor* or tumour*) OR SU (neoplasm* or cancer* or oncolog* or malignan* or tumor* or tumour*)	Search modes - Proximity	Interface - EBSCOhost Research Databases<br>Search Screen - Advanced Search<br>Database - CINAHL Complete	Display
S7	(MH "Neoplasms+") OR (MH "Cancer Patients") OR (MH "Cancer Survivors")	Search modes - Proximity	Interface - EBSCOhost Research Databases<br>Search Screen - Advanced Search<br>Database - CINAHL Complete	Display
S6	S1 OR S2 OR S3 OR S4 OR S5	Search modes - Proximity	Interface - EBSCOhost Research Databases<br>Search Screen - Advanced Search<br>Database - CINAHL Complete	Display
S5	TI adult* OR AB adult* OR SU adult*	Search modes - Proximity	Interface - EBSCOhost Research Databases	Display

			Search Screen - Advanced Search<br>Database - CINAHL Complete	
S4	TI ((highschool* or college* or university or "secondary school") N2 student*)) OR AB ((highschool* or college* or university or "secondary school") N2 student*)) OR SU ((highschool* or college* or university or "secondary school") N2 student*))	Search modes - Proximity	Interface - EBSCOhost Research Databases<br>Search Screen - Advanced Search<br>Database - CINAHL Complete	Display
S3	TI ((young or emerging) N1 (adult* or person* or individual* or people* or population* or man or men or wom?n)) OR AB ((young or emerging) N1 (adult* or person* or individual* or people* or population* or man or men or wom?n)) OR SU ((young or emerging) N1 (adult* or person* or individual* or people* or population* or man or men or wom?n))	Search modes - Proximity	Interface - EBSCOhost Research Databases<br>Search Screen - Advanced Search<br>Database - CINAHL Complete	Display
S2	TI ((teen* or adolecen* or youth or AYA)) OR AB ((teen* or adolecen* or youth or AYA)) OR SU ((teen* or adolecen* or youth or AYA))	Search modes - Proximity	Interface - EBSCOhost Research Databases<br>Search Screen - Advanced Search<br>Database - CINAHL Complete	Display
S1	(MH "Adolescence") OR (MH "Adult") OR (MH "Young Adult")	Search modes - Proximity	Interface - EBSCOhost Research Databases<br>Search Screen - Advanced Search<br>Database - CINAHL Complete	Display
