# Supplementary material for: The Experiences of Adolescents and Young Adults with Digital Supportive Care Interventions for Cancer: A Systematic Review of Qualitative Studies
Source: Cancers (Basel). 2025 Feb 21;17(5):736. doi: 10.3390/cancers17050736 (PMC11899503; doi:10.3390/cancers17050736)
Supplement: Supplementary file 1 [file cancers-17-00736-s001.zip › Supplementary Table S2 (SS_PsycINFO).pdf]

EBSCOhost

#	Query	Limiters/Expanders	Last Run Via	Results
S50	S9 AND S12 AND S24 AND S40 AND S47	Limiters - Publication Year: 2000-2024<br>Search modes - Proximity	Interface - EBSCOhost Research Databases<br>Search Screen - Advanced Search<br>Database - APA PsycInfo	Display
S49	S9 AND S12 AND S24 AND S40 AND S47	Limiters - Publication Year: 2000-2024<br>Search modes - Proximity	Interface - EBSCOhost Research Databases<br>Search Screen - Advanced Search<br>Database - APA PsycInfo	Display
S48	S9 AND S12 AND S24 AND S40 AND S47	Search modes - Proximity	Interface - EBSCOhost Research Databases<br>Search Screen - Advanced Search<br>Database - APA PsycInfo	Display
S47	S41 OR S42 OR S43 OR S44 OR S45 OR S46	Search modes - Proximity	Interface - EBSCOhost Research Databases<br>Search Screen - Advanced Search<br>Database - APA PsycInfo	Display
S46	TI (experience* or impression* or evaluat*) OR AB (experience* or impression* or evaluat*) OR KW (experience* or impression* or evaluat*)	Search modes - Proximity	Interface - EBSCOhost Research Databases<br>Search Screen - Advanced Search<br>Database - APA PsycInfo	Display
S45	TI ((field N1 work) or fieldwork or (focus N1 (group or groups)) or (key N1 informant*)) OR AB ((field N1 work) or fieldwork or (focus N1 (group or groups)) or (key	Search modes - Proximity	Interface - EBSCOhost Research Databases<br>Search Screen - Advanced Search<br>Database - APA PsycInfo	Display

	N1 informant*)) OR KW ((field N1 work) or fieldwork or (focus N1 (group or groups)) or (key N1 informant*))			
S44	TI (ethnograph* OR "grounded theory" or "content analysis" or "framework analysis" or "thematic analysis") OR AB (ethnograph* OR "grounded theory" or "content analysis" or "framework analysis" or "thematic analysis") OR KW (ethnograph* OR "grounded theory" or "content analysis" or "framework analysis" or "thematic analysis")	Search modes - Proximity	Interface - EBSCOhost Research Databases<br>Search Screen - Advanced Search<br>Database - APA PsycInfo	Display
S43	TI (((face OR f2f OR "face-to-face" OR guide* OR depth OR indepth OR "in-depth" OR informal OR semistructured OR "semi-structured" OR structured or unstructured) N3 (discussion* OR interview* OR questionnaire*))) OR AB (((face OR f2f OR "face-to-face" OR guide* OR depth OR indepth OR "in-depth" OR informal OR semistructured OR "semi-structured" OR structured or unstructured) N3 (discussion* OR interview* OR questionnaire*))) OR KW (((face OR f2f OR "face-to-face" OR guide* OR depth OR indepth OR "in-depth" OR informal OR semistructured OR "semi-	Search modes - Proximity	Interface - EBSCOhost Research Databases<br>Search Screen - Advanced Search<br>Database - APA PsycInfo	Display

	structured" OR structured or unstructured) N3 (discussion* OR interview* OR questionnaire*)))			
S42	TI (qualitative OR interview* OR "focus group*" OR diary OR open-ended OR narrative) OR AB (qualitative OR interview* OR "focus group*" OR diary OR open-ended OR narrative) OR KW (qualitative OR interview* OR "focus group*" OR diary OR open-ended OR narrative)	Search modes - Proximity	Interface - EBSCOhost Research Databases<br>Search Screen - Advanced Search<br>Database - APA PsycInfo	Display
S41	DE "Focus Group Interview" OR DE "Qualitative Methods" OR DE "Focus Group" OR DE "Grounded Theory" OR DE "Interpretative Phenomenological Analysis" OR DE "Narrative Analysis" OR DE "Semi-Structured Interview" OR DE "Thematic Analysis" OR DE "Interviews" OR DE "Observation Methods" OR DE "Phenomenology" OR DE "Qualitative Measures"	Search modes - Proximity	Interface - EBSCOhost Research Databases<br>Search Screen - Advanced Search<br>Database - APA PsycInfo	Display
S40	S25 OR S26 OR S27 OR S28 OR S29 OR S30 OR S31 OR S32 OR S33 OR S34 OR S35 OR S36 OR S37 OR S38 OR S39	Search modes - Proximity	Interface - EBSCOhost Research Databases<br>Search Screen - Advanced Search<br>Database - APA PsycInfo	Display
S39	DE "Sleep" OR DE "Sleep Treatment" OR DE	Search modes - Proximity	Interface - EBSCOhost Research Databases	Display

	"Insomnia OR DE "Major Depression" AND DE "Anxiety" OR DE "Death Anxiety" OR DE "Health Anxiety"		Search Screen - Advanced Search<br>Database - APA PsycInfo	
S38	TI ((spiritual OR pastoral) N1 (care or therap*)) OR AB ((spiritual OR pastoral) N1 (care or therap*)) OR KW ((spiritual OR pastoral) N1 (care or therap*))	Search modes - Proximity	Interface - EBSCOhost Research Databases<br>Search Screen - Advanced Search<br>Database - APA PsycInfo	Display
S37	TI (exercise* OR nutrition OR diet OR physical activit* OR "mental health" OR counsel?ing OR psychotherap* OR physical therap* OR physiotherap* OR "mind-body" OR "Social support" OR "supportive care" OR "sexual health" OR "pain management" OR palliative) OR AB (exercise* OR nutrition OR diet OR physical activit* OR "mental health" OR counsel?ing OR psychotherap* OR physical therap* OR physiotherap* OR "mind-body" OR "Social support" OR "supportive care" OR "sexual health" OR "pain management" OR palliative) OR KW (exercise* OR nutrition OR diet OR physical activit* OR "mental health" OR counsel?ing OR psychotherap* OR physical therap* OR physiotherap* OR "mind-	Search modes - Proximity	Interface - EBSCOhost Research Databases<br>Search Screen - Advanced Search<br>Database - APA PsycInfo	Display

	body" OR "Social support" OR "supportive care" OR "sexual health" OR "pain management" OR palliative)			
S36	DE "Palliative Care" OR DE "Symptoms Based Treatment"	Search modes - Proximity	Interface - EBSCOhost Research Databases<br>Search Screen - Advanced Search<br>Database - APA PsycInfo	Display
S35	DE "Pain Management"	Search modes - Proximity	Interface - EBSCOhost Research Databases<br>Search Screen - Advanced Search<br>Database - APA PsycInfo	Display
S34	DE "Sexual Health"	Search modes - Proximity	Interface - EBSCOhost Research Databases<br>Search Screen - Advanced Search<br>Database - APA PsycInfo	Display
S33	DE "Social Support"	Search modes - Proximity	Interface - EBSCOhost Research Databases<br>Search Screen - Advanced Search<br>Database - APA PsycInfo	Display
S32	DE "Spiritually Oriented Therapy" OR DE "Spiritual Care"	Search modes - Proximity	Interface - EBSCOhost Research Databases<br>Search Screen - Advanced Search<br>Database - APA PsycInfo	Display
S31	DE "Mind Body Therapy" OR DE "Relaxation Therapy" OR DE "Mindfulness-Based Stress Reduction" OR DE "Mindfulness-Based Interventions" OR DE "Mindfulness Meditation"	Search modes - Proximity	Interface - EBSCOhost Research Databases<br>Search Screen - Advanced Search<br>Database - APA PsycInfo	Display
S30	DE "Psychosocial Interventions"	Search modes - Proximity	Interface - EBSCOhost Research Databases	Display

			Search Screen - Advanced Search<br>Database - APA PsycInfo	
S29	DE "Mental Health" OR DE "Youth Mental Health" OR DE "Emotional Health" OR DE "Self-Care" OR DE "Distress" OR DE "Health Related Quality of Life"	Search modes - Proximity	Interface - EBSCOhost Research Databases<br>Search Screen - Advanced Search<br>Database - APA PsycInfo	Display
S28	DE "Nutrition" OR DE "Dietary Treatment"	Search modes - Proximity	Interface - EBSCOhost Research Databases<br>Search Screen - Advanced Search<br>Database - APA PsycInfo	Display
S27	DE "Exercise" OR DE "Exercise Therapy"	Search modes - Proximity	Interface - EBSCOhost Research Databases<br>Search Screen - Advanced Search<br>Database - APA PsycInfo	Display
S26	DE "Counseling" OR DE "Group Counseling" OR DE "Pastoral Counseling" OR DE "Psychotherapeutic Counseling" OR DE "Family Therapy" OR DE "Rehabilitation Counseling" OR DE "Counseling Psychology" OR DE "Mental Health Services" OR DE "College Mental Health Services" OR DE "Community Mental Health Services" OR DE "Mental Health Programs" OR DE "Psychological First Aid" OR DE "Support Groups"	Search modes - Proximity	Interface - EBSCOhost Research Databases<br>Search Screen - Advanced Search<br>Database - APA PsycInfo	Display
S25	DE "Psychotherapy" OR DE "Adlerian	Search modes - Proximity	Interface - EBSCOhost Research Databases	Display

Psychotherapy" OR DE "Adolescent Psychotherapy" OR DE "Affirmative Therapy" OR DE "Analytical Psychotherapy" OR DE "Autogenic Training" OR DE "Brief Psychotherapy" OR DE "Brief Relational Therapy" OR DE "Child Psychotherapy" OR DE "Client Centered Therapy" OR DE "Compassion Focused Therapy" OR DE "Couples Therapy" OR DE "Eclectic Psychotherapy" OR DE "Emotion Focused Therapy" OR DE "Existential Therapy" OR DE "Experiential Psychotherapy" OR DE "Expressive Psychotherapy" OR DE "Eye Movement Desensitization Therapy" OR DE "Feminist Therapy" OR DE "Geriatric Psychotherapy" OR DE "Gestalt Therapy" OR DE "Group Psychotherapy" OR DE "Guided Imagery" OR DE "Humanistic Psychotherapy" OR DE "Hypnotherapy" OR DE "Individual Psychotherapy" OR DE "Insight Therapy" OR DE "Integrative Psychotherapy" OR DE "Interpersonal Psychotherapy" OR DE "Logotherapy" OR DE "Metacognitive Therapy"

Search Screen - Advanced Search
Database - APA PsycInfo

OR DE "Narrative Therapy" OR DE "Network Therapy" OR DE "Personal Therapy" OR DE "Persuasion Therapy" OR DE "Positive Psychology Therapy" OR DE "Primal Therapy" OR DE "Psychoanalysis" OR DE "Psychodrama" OR DE "Psychodynamic Psychotherapy" OR DE "Psychotherapeutic Counseling" OR DE "Psychotherapeutic Techniques" OR DE "Rational Emotive Behavior Therapy" OR DE "Reality Therapy" OR DE "Relationship Therapy" OR DE "Solution Focused Therapy" OR DE "Spiritually Oriented Therapy" OR DE "Strategic Therapy" OR DE "Supportive Psychotherapy" OR DE "Transactional Analysis" OR DE "Psychotherapeutic Counseling" OR DE "Family Therapy" OR DE "Psychotherapeutic Techniques" OR DE "Active Listening" OR DE "Autogenic Training" OR DE "Brief Relational Therapy" OR DE "Centering" OR DE "Cotherapy" OR DE "Dream Analysis" OR DE "Empty Chair Technique" OR DE "Ericksonian Psychotherapy" OR DE

	"Free Association" OR DE "Guided Imagery" OR DE "Life Review" OR DE "Mirroring" OR DE "Morita Therapy" OR DE "Motivational Interviewing" OR DE "Mutual Storytelling Technique" OR DE "Network Therapy" OR DE "Paradoxical Techniques" OR DE "Psychodrama" OR DE "Self-Affirmation" OR DE "Cognitive Therapy" OR DE "Pastoral Counseling" OR DE "Recreation Therapy"			
S24	S13 OR S14 OR S15 OR S16 OR S17 OR S18 OR S19 OR S20 OR S21 OR S22 OR S23	Search modes - Proximity	Interface - EBSCOhost Research Databases<br>Search Screen - Advanced Search<br>Database - APA PsycInfo	Display
S23	TI (email OR "e-mail" OR "electronic mail" OR "text messaging" OR "Social Media" OR Facebook OR Instagram OR YouTube OR Zoom OR "online community" OR "online social network" OR internet OR website*) OR AB (email OR "e-mail" OR "electronic mail" OR "text messaging" OR "Social Media" OR Facebook OR Instagram OR YouTube OR Zoom OR "online community" OR "online social network" OR internet OR website*) OR KW (email OR "e-mail" OR "electronic mail" OR "text messaging" OR	Search modes - Proximity	Interface - EBSCOhost Research Databases<br>Search Screen - Advanced Search<br>Database - APA PsycInfo	Display

	"Social Media" OR Facebook OR Instagram OR YouTube OR Zoom OR "online community" OR "online social network" OR internet OR website*)			
S22	DE "Computer Mediated Communication" OR DE "Electronic Communication" OR DE "Text Messaging" OR DE "Social Media" OR DE "Internet" OR DE Internet Usage" OR DE "Online Community" OR DE "Online Social Networks" OR DE "Websites"	Search modes - Proximity	Interface - EBSCOhost Research Databases<br>Search Screen - Advanced Search<br>Database - APA PsycInfo	Display
S21	TI ((Wearable Device*) OR (Fitbit) OR (activity N1 (monitor* or tracker*)) OR (Mobile Phone*) OR (Smartphone*) OR (Wireless Technolog*)) OR AB ((Wearable Device*) OR (Fitbit) OR (activity N1 (monitor* or tracker*)) OR (Mobile Phone*) OR (Smartphone*) OR (Fitbit) OR (activity N1 (monitor* or tracker*)) OR (Wireless Technolog*)) OR KW ((Wearable Device*) OR (Fitbit) OR (activity N1 (monitor* or tracker*)) OR (Mobile phone*) OR (Smartphone*) OR (Wireless Technolog*))	Search modes - Proximity	Interface - EBSCOhost Research Databases<br>Search Screen - Advanced Search<br>Database - APA PsycInfo	Display
S20	DE "Wearable Devices" OR DE "Mobile Phones" OR DE "Mobile Devices" OR DE "Smartphones"	Search modes - Proximity	Interface - EBSCOhost Research Databases<br>Search Screen - Advanced	Display

	OR DE "Wireless Technologies"		Search<br>Database - APA PsycInfo	
S19	TI ((Computer N1 (Application* OR Simulation OR Software)) OR (app OR apps OR Digital technolog* OR Mobile application*)) OR AB ((Computer N1 (Application* OR Simulation OR Software)) OR (app OR apps OR Digital technolog* OR Mobile application*)) OR KW ((Computer N1 (Application* OR Simulation OR Software)) OR (app OR apps OR Digital technolog* OR Mobile application*))	Search modes - Proximity	Interface - EBSCOhost Research Databases<br>Search Screen - Advanced Search<br>Database - APA PsycInfo	Display
S18	DE "Computer Applications" OR DE "Digital Technology" OR DE "Mobile Applications" OR DE "Computer Games" OR DE "Computer Simulation" OR DE "Computer Software" OR DE "Digital Gaming" OR DE "Digital Game-Based Learning" OR DE "Virtual Reality" OR DE "Augmented Reality" OR DE "Virtual Environment"	Search modes - Proximity	Interface - EBSCOhost Research Databases<br>Search Screen - Advanced Search<br>Database - APA PsycInfo	Display
S17	TI ((electronic or mobile or digital) N1 device*) OR AB ((electronic or mobile or digital) N1 device*) OR KW ((electronic or mobile or digital) N1 device*)	Search modes - Proximity	Interface - EBSCOhost Research Databases<br>Search Screen - Advanced Search<br>Database - APA PsycInfo	Display

S16	TI ((virtual OR remote OR digital OR mobile OR online OR hybrid) N3 (care OR health* OR intervention* OR therapy OR counsel#ing)) OR AB ((virtual or remote or digital or mobile or online or hybrid) N3 (care OR health* OR intervention* OR therapy OR counsel#ing) OR KW ((virtual or remote or digital or mobile or online or hybrid) N3 (care OR health* OR intervention* OR therapy OR counsel#ing))	Search modes - Proximity	Interface - EBSCOhost Research Databases<br>Search Screen - Advanced Search<br>Database - APA PsycInfo	Display
S15	TI (e-health OR ehealth OR m-health OR telehealth OR telemedicine) OR AB (e-health OR ehealth OR m-health OR telehealth OR telemedicine) OR KW (e-health OR ehealth OR m-health OR telehealth OR telemedicine)	Search modes - Proximity	Interface - EBSCOhost Research Databases<br>Search Screen - Advanced Search<br>Database - APA PsycInfo	Display
S14	DE "Digital Interventions" OR DE "Mobile Health" OR DE "Electronic Health Services" OR DE "Telemedicine" OR DE "Digital Mental Health Resources" OR DE "Digital Health Resources" OR DE "Online Therapy" OR DE "Telepsychology" OR DE "Computer Assisted Therapy"	Search modes - Proximity	Interface - EBSCOhost Research Databases<br>Search Screen - Advanced Search<br>Database - APA PsycInfo	Display
S13	DE "Teleconferencing" OR DE "Video-Based	Search modes - Proximity	Interface - EBSCOhost Research Databases	Display

	Interventions" OR DE "Digital Video" OR DE "Videoconferencing"		Search Screen - Advanced Search<br>Database - APA PsycInfo	
S12	S10 OR S11	Search modes - Proximity	Interface - EBSCOhost Research Databases<br>Search Screen - Advanced Search<br>Database - APA PsycInfo	Display
S11	TI (neoplasm* or cancer* or oncolog* or malignan* or tumor* or tumour*) OR AB (neoplasm* or cancer* or oncolog* or malignan* or tumor* or tumour*) OR KW (neoplasm* or cancer* or oncolog* or malignan* or tumor* or tumour*)	Search modes - Proximity	Interface - EBSCOhost Research Databases<br>Search Screen - Advanced Search<br>Database - APA PsycInfo	Display
S10	DE "Neoplasms" OR DE "Benign Neoplasms" OR DE "Breast Neoplasms" OR DE "Endocrine Neoplasms" OR DE "Leukemias" OR DE "Melanoma" OR DE "Metastasis" OR DE "Nervous System Neoplasms" OR DE "Terminal Cancer"	Search modes - Proximity	Interface - EBSCOhost Research Databases<br>Search Screen - Advanced Search<br>Database - APA PsycInfo	Display
S9	S1 OR S2 OR S3 OR S4 OR S5 OR S6 OR S7 OR S8	Search modes - Proximity	Interface - EBSCOhost Research Databases<br>Search Screen - Advanced Search<br>Database - APA PsycInfo	Display
S8	TI adult* OR AB adult* OR KW adult*	Search modes - Proximity	Interface - EBSCOhost Research Databases<br>Search Screen - Advanced Search<br>Database - APA PsycInfo	Display

S7	TI ((highschool* or college* or university or "secondary school") N2 student*) OR AB ((highschool* or college* or university or "secondary school") N2 student*) OR KW ((highschool* or college* or university or "secondary school") N2 student*)	Search modes - Proximity	Interface - EBSCOhost Research Databases<br>Search Screen - Advanced Search<br>Database - APA PsycInfo	Display
S6	TI ((young or emerging) N1 (adult* or person* or individual* or people* or population* or man or men or wom?n)) OR AB ((young or emerging) N1 (adult* or person* or individual* or people* or population* or man or men or wom?n)) OR KW ((young or emerging) N1 (adult* or person* or individual* or people* or population* or man or men or wom?n))	Search modes - Proximity	Interface - EBSCOhost Research Databases<br>Search Screen - Advanced Search<br>Database - APA PsycInfo	Display
S5	TI (teen* or adolecen* or youth or AYA) OR AB (teen* or adolecen* or youth or AYA) OR KW (teen* or adolecen* or youth or AYA)	Search modes - Proximity	Interface - EBSCOhost Research Databases<br>Search Screen - Advanced Search<br>Database - APA PsycInfo	Display
S4	DE "Adolescent Attitudes"	Search modes - Proximity	Interface - EBSCOhost Research Databases<br>Search Screen - Advanced Search<br>Database - APA PsycInfo	Display
S3	DE "Adult Attitudes"	Search modes - Proximity	Interface - EBSCOhost Research Databases<br>Search Screen - Advanced	Display

			Search<br>Database - APA PsycInfo	
S2	DE "Emerging Adulthood"	Search modes - Proximity	Interface - EBSCOhost Research Databases<br>Search Screen - Advanced Search<br>Database - APA PsycInfo	Display
S1	DE "Late Adolescence"	Search modes - Proximity	Interface - EBSCOhost Research Databases<br>Search Screen - Advanced Search<br>Database - APA PsycInfo	Display
