# Supplementary material for: The Experiences of Adolescents and Young Adults with Digital Supportive Care Interventions for Cancer: A Systematic Review of Qualitative Studies
Source: Cancers (Basel). 2025 Feb 21;17(5):736. doi: 10.3390/cancers17050736 (PMC11899503; doi:10.3390/cancers17050736)
Supplement: Supplementary file 1 [file cancers-17-00736-s001.zip › Supplementary Table S3 (SS_EMBASE).pdf]

Table S3: Search Strategy for EMBASE

Records before removing duplicates: 3,470
Original searches (exactly as executed):

#1 adolescent/
#2 adolescent*.ti,ab,hw,kf.
#3 (teen* or youth* or AYA).ti,ab,hw,kf.
#4 Young Adult/
#5 ((young or emerging) adj2 (adult* or person* or individual* or people* or population* or man or men or wom#n)).ti,ab,hw,kf.
#6 ((highschool* or college* or university or "secondary school") adj2 student*).ti,ab,hw,kf.
#7 adult/
#8 adult*.ti,ab,hw,kf.
#9 or/1-8
#10 exp malignant neoplasm/
#11 cancer survivor/ or cancer patient/
#12 (neoplasm* or cancer* or oncolog* or malignan* or tumor* or tumour*).ti,ab,hw,kf.
#13 or/10-12
#14 videoconferencing/
#15 teleconsultation/ or telemedicine/ or electronic consultation/
#16 telehealth/
#17 telerehabilitation/
#18 (e-health or ehealth or m-health or telehealth or telemedicine).ti,ab,hw,kf.
#19 ((virtual or remote or digital or mobile or online or hybrid) adj3 (care or health* or intervention*)).ti,ab,hw,kf.
#20 ((electronic or mobile or digital) adj device*).ti,ab,hw,kf.
#21 online social network/ or social network/
#22 attitude to computers/
#23 exp Internet/
#24 telepsychotherapy/ or web-based intervention/ or telepsychiatry/ or telepsychology/
#25 exp mobile application/
#26 mobile phone/ or smartphone/ or text messaging/
#27 (app or apps or smartphone* or "social media" or internet or "text message*" or web-based).ti,ab,hw,kf.
#28 (facebook or zoom or fitbit or instagram).ti,ab,hw,kf.
#29 physiologic monitoring/
#30 exp activity tracker/ or exp smart watch/
#31 (activity adj1 (monitor* or tracker*)).ti,ab,hw,kf.
#32 smartwatch.ti,ab,hw,kf.
#33 or/14-32
#34 exp exercise/
#35 exp kinesiotherapy/
#36 exp diet therapy/

#37 exp diet/
#38 mental health/ or psychological well-being/ or psychological resilience/
#39 "quality of life"/
#40 psychological counseling/ or exp psychological care/
#41 exp counseling/
#42 exp psychotherapy/
#43 psychosocial care/ or mental health care/
#44 psychosocial intervention/
#45 exp yoga/ or exp mindfulness/ or exp meditation/
#46 spiritual care/ or spiritual healing/ or pastoral care/
#47 social support/ or community support/ or emotional support/
#48 sexual health/
#49 analgesia/
#50 cancer palliative therapy/
#51 exp rehabilitation/
#52 physiotherapy/
#53 (exercise* or nutrition or diet or "physical activit*" or "mental health" or counsel?ing or psychotherap* or palliative or "physical therap*" or physiotherap* or "mind-body" or "sexual health").ti,ab,hw,kf.
#54 (spiritual adj1 (care or therap*)).ti,ab,hw,kf.
#55 (pastoral adj1 (care or therap*)).ti,ab,hw,kf.
#56 (pain adj2 manage*).ti,ab,hw,kf.
#57 (supportive adj1 care).ti,ab,hw,kf.
#58 exp sleep/
#59 cancer fatigue/ or fatigue/
#60 insomnia/
#61 anxiety/ or "fear of death"/
#62 depression/
#63 (sleep* or fatigue or insomnia or anxiety or depress*).ti,ab,hw,kf.
#64 or/34-63
#65 exp qualitative research/
#66 grounded theory/
#67 qualitative.ti,ab,hw,kf.
#68 ((face or f2f or "face-to-face" or guide* or depth or indepth or "in-depth" or informal or semistructured or "semi-structured" or structured or unstructured) adj3 (discussion* or interview* or questionnaire*)).ti,ab,hw,kf.
#69 (ethnograph* or (field adj1 work) or fieldwork or (focus adj1 (group or groups)) or (key adj1 informant*) or qualitative).ti,ab,hw,kf.
#70 ("grounded theory" or "content analysis" or "framework analysis" or "thematic analysis").ti,ab,hw,kf.
#71 (experience* or impression* or evaluat*).ti,ab,hw,kf.
#72 exp interview/
#73 narrative/

#**74** or/65-73
#**75** 9 and 13 and 33 and 64 and 74
#**76** limit 75 to (english language and yr="2000 -Current")
#**77** limit 76 to conference abstract
#**78** 76 not 77
#**79** from 78 keep 1-3378
