# Supplementary material for: The Experiences of Adolescents and Young Adults with Digital Supportive Care Interventions for Cancer: A Systematic Review of Qualitative Studies
Source: Cancers (Basel). 2025 Feb 21;17(5):736. doi: 10.3390/cancers17050736 (PMC11899503; doi:10.3390/cancers17050736)
Supplement: Supplementary file 1 [file cancers-17-00736-s001.zip › Supplementary Table S5 (Coding Framework).pdf]

Table S5: Coding framework to capture the experiences of AYAs with cancer when using digital supportive care interventions

**Codes in Nvivo**				**Translated Themes**	
**Concept**	**Level 1**	**Level 2**	**Level 3**	**Descriptive Themes**	**Sub-themes**
**FACILITATORS**<br>*Facilitators of using digital health interventions*	**C1.** Preferences<br>*Identifying one's preferences*	**C1.1.** Content<br>*Amenable to one's needs (e.g. age or cancer type-based content/module)*		**T1.** Positive Experiences with Intervention Attributes	**T1.1.** Appropriate Content
		**C1.2.** Choice<br>*Flexibility (e.g., from my own home, timing of sessions, when to use intervention, camera on/off)*			**T1.2.** Flexible Choice
		**C1.3.** Technology<br>*Usability of the provided technology*			**T1.3.** Seamless Technology
		**C1.4.** Desirable Environment<br>*(i.e., safety, privacy, inclusivity)*			**T1.4.** Inclusive Environment
	**C2.** Health & Well-being<br>*Benefits of using the intervention*	**C2.1.** Connections & Communication<br>*Forming meaningful connections and learning effective communication*	**C2.1.1.** HCPs<br>*With doctors, nurses, therapists, or other care providers*	**T2.** Facilitated Intervention Outcomes: Health & Well-being	**T2.1.** Enhanced Connections and Communication Skills
			**C2.1.2.** Family<br>*With partners, children, parents, etc.*		
			**C2.1.3.** Peers<br>*With friends, colleagues, and peers outside and/or inside the interventions*		
		**C2.2.** Health<br>*Improvements in one's overall health*	**C2.2.1.** Physical health<br>*Exercise/nutrition routines, becoming more active, building up stamina, etc.*		**T2.2.** Improved Physical Health
			**C2.2.2.** Psychological health<br>*Beneficial to mental health (e.g. meditation, mindfulness, yoga, music), alleviating mental health challenges (e.g. stress, isolation, sleep etc.)*		**T2.3.** Improved Psychological Health

		**C2.3.** Becoming Autonomous<br>*Freedom to act and to make one's own choices.*	**C2.3.1.** Motivation & Empowerment<br>*Limited form of control – own set of rules, procedures, boundaries; New skills learned; self-manage symptoms; self-reflect; motivated to move forward*		**T2.4.** Autonomy
**BARRIERS**<br>*Barriers to using digital health interventions*	**C3.** Missing Preferences<br>*Does not incorporate one's preferences*	**C3.1.** Poor Content<br>*Cannot relate to the module or content of the intervention (not tailored, not need-specific or too generic)*		**T3.** Negative Experiences with Intervention Attributes	**T3.1.** Generic Content
		**C3.2.** Lack of Choice<br>*Frequent measurements, notifications, demanding time; inflexible with time or location*			**T3.2.** Limited Choices
		**C3.3.** Technological Issues<br>*Issues/glitches with the technology provided*			**T3.3.** Faulty Technology
		**C3.4.** Unfavorable Environment<br>*Lack of inclusivity in the intervention (e.g., too closed off, anonymity, feeling detached from the intervention, etc.)*			**T3.4.** Unfavorable Environment
	**C4.** Lacking in contribution to Health and Well-being	**C4.1.** Missing connection and communication<br>*The desire for more meaningful connections*	**C4.1.1.** HCPs<br>*With healthcare providers*	**T4.** Hindered Intervention Outcomes: Health and Well-being	**T4.1**. Lack of Communication and Connection
			**C4.1.2.** Family<br>*With family members/partners*		
			**C4.1.3.** Peers<br>*With peers*		
		**C4.2.Triggering**<br>*The intervention brings up strong feelings, avoidance, or anything that contributes to mental health challenges.*			**T4.2.** Triggering
