# Supplementary material for: The Experiences of Adolescents and Young Adults with Digital Supportive Care Interventions for Cancer: A Systematic Review of Qualitative Studies
Source: Cancers (Basel). 2025 Feb 21;17(5):736. doi: 10.3390/cancers17050736 (PMC11899503; doi:10.3390/cancers17050736)
Supplement: Supplementary file 1 [file cancers-17-00736-s001.zip › Supplementary Table S6 (CASP).pdf]

TABLE S6: CASP CHECKLIST

Q1: Was there a clear statement of the aims of the research?
Q2: Is a qualitative methodology appropriate?
Q3: Was the research design appropriate to address the aims of the research?
Q4. Was the recruitment strategy appropriate to the aims of the research?
Q5. Was the data collected in a way that addressed the research issue?

Q6: Has the relationship between researcher and participants been adequately considered?
Q7: Have ethical issues been taken into consideration?
Q8: Was the data analysis sufficiently rigorous?
Q9: Is there a clear statement of findings?
Q10: How valuable is the research?

*Assessment Result= Research team's ranking of the studies
If all yes = robust
If 1 no/can't tell = moderately strong
If 2-3 no/can't tell (combined) = moderate
If 4+ no/can't tell (combined) = weak

**First Author**	**Section A**						**Section B**			**Section C**	**Assessment Ranking***	**Appraisal Summary**
Last Name	***Q1.***	***Q2.***	***Q3.***	***Q4.***	***Q5***	***Q6.***	***Q7.***	***Q8.***	***Q9.***	***Q10***		***Key Comments***
**Barnes**	Yes	Yes	No	Can't Tell	Yes	Can't Tell	Yes	Yes	Yes	Yes	Moderate	Strong analysis, but have not met a few criteria, weak methods.
**Conduit**	Yes	Yes	Yes	Yes	Yes	Yes	Yes	Yes	Yes	Yes	Robust	They explored all the CASP criteria. Overall, very sound design
**Donovan**	Yes	Yes	No	Yes	Yes	Can't Tell	Yes	No	Yes	Yes	Moderate	Overall, it is lacking in the design and description of the analysis.
**Erikson**	Yes	Yes	Yes	Yes	Yes	Yes	Can't Tell	Yes	Yes	Yes	Moderately strong	Sound design and provide an outlook on their qualitative analysis steps. With minor clarifications is the methods, this can be recommended or replicated for wider usage.
**Fergus (2014)**	Yes	Yes	No	Yes	Yes	Yes	Yes	No	Yes	Yes	Moderate	The pilot study design was decent, with a few criteria lacking justification.
**Fergus (2017)**	Yes	Yes	Yes	Yes	Can't Tell	Can't Tell	Yes	Yes	Yes	Can't Tell	Moderate	The study has a good design; however, some criteria were not adequately explored. Some of the limitations mentioned by the authors make the intervention challenging to replicate or recommend
**Greer**	Yes	Can't Tell	No	Yes	Yes	No	Yes	No	Can't Tell	Yes	Weak	Weak data collection, analysis, and missing thematic analysis. The intervention may need further exploration

TABLE S6: CASP CHECKLIST

Q1: Was there a clear statement of the aims of the research?
Q2: Is a qualitative methodology appropriate?
Q3: Was the research design appropriate to address the aims of the research?
Q4. Was the recruitment strategy appropriate to the aims of the research?
Q5. Was the data collected in a way that addressed the research issue?
Q6: Has the relationship between researcher and participants been adequately considered?
Q7: Have ethical issues been taken into consideration?
Q8: Was the data analysis sufficiently rigorous?
Q9: Is there a clear statement of findings?
Q10: How valuable is the research?

*Assessment Result= Research team's ranking of the studies
If all yes = robust
If 1 no/can't tell = moderately strong
If 2-3 no/can't tell (combined) = moderate
If 4+ no/can't tell (combined) = weak

**Hanghøj**	Yes	Yes	Yes	Yes	Yes	Can't Tell	Yes	Yes	Yes	Yes	Moderately strong	Well-designed paper with adequate justifications for the methods. With few modifications, the intervention can be recommended.
**Heiniger**	Yes	No	No	Yes	Can't Tell	No	Yes	Yes	Yes	Yes	Weak	While the intervention is sound, the qualitative part is relatively weak.
**Jibb**	Yes	Yes	Yes	Yes	Yes	Can't Tell	Yes	Yes	Yes	Yes	Moderately strong	A strong paper that explores most of the criteria and can be recommended or replicated.
**Lichiello**	Yes	Yes	No	Yes	Yes	Can't Tell	Yes	Can't Tell	Yes	Yes	Moderate	Well-designed study, but lacking in research design justification and critical examination of roles and biases
**Markwardt**	Yes	Yes	Yes	Yes	Yes	Can't Tell	Yes	Yes	Yes	Yes	Moderately strong	Sounds methods with adequate justification. It can be replication or recommended.
**Melton**	Yes	Yes	No	Can't Tell	Yes	Can't Tell	Yes	No	Yes	Yes	Weak	Lack of critical analysis of biases and roles, as well as design justifications. The study was not sufficiently rigorous.
**Mendoza**	Yes	No	No	Yes	Yes	Can't Tell	Can't Tell	No	Yes	No	Weak	The impact of the qualitative findings was inadequately explored as the quantitative portion was more of the focus of the study
**Micaux**	Yes	Yes	Yes	Yes	Yes	Can't Tell	Yes	Yes	Yes	Yes	Moderately strong	An intervention can be designed based on what is learned from this intervention.
**Miropolsky**	Yes	Yes	No	Yes	Yes	No	Can't Tell	Yes	Yes	No	Moderate	The methodology can be improved. Overall, very thorough with thematic analysis.
**Perumbil**	Yes	Yes	No	Can't Tell	Yes	No	Yes	Yes	Yes	Yes	Moderate	Lack of explanation of research design and justifications for some of the methods.

TABLE S6: CASP CHECKLIST

Q1: Was there a clear statement of the aims of the research?
Q2: Is a qualitative methodology appropriate?
Q3: Was the research design appropriate to address the aims of the research?
Q4. Was the recruitment strategy appropriate to the aims of the research?
Q5. Was the data collected in a way that addressed the research issue?

Q6: Has the relationship between researcher and participants been adequately considered?
Q7: Have ethical issues been taken into consideration?
Q8: Was the data analysis sufficiently rigorous?
Q9: Is there a clear statement of findings?
Q10: How valuable is the research?

*Assessment Result= Research team's ranking of the studies
If all yes = robust
If 1 no/can't tell = moderately strong
If 2-3 no/can't tell (combined) = moderate
If 4+ no/can't tell (combined) = weak

**Phillips**	Yes	Yes	Can't Tell	Yes	Yes	Yes	Yes	Yes	Yes	Yes	Moderately strong	The research design is sound; however, it is very COVID-specific. The intervention may not be suitable in the long term as per the participant's experiences.
**Poort**	Yes	Yes	Yes	Yes	Yes	Yes	Yes	Yes	Yes	No	Moderately strong	A well-planned qualitative section. Although the app did not meet the feasibility criteria, it gained valuable insights. It can be recommended for wider usage.
**Price (2021)**	Yes	Yes	No	Yes	Yes	No	Yes	Yes	Yes	Can't Tell	Moderate	Presents a thorough description of the qualitative portion and how the analysis was conducted. Some of the criteria are missing; hence, we are unable to ascertain feasibility.
**Price (2022)**	Yes	Yes	Yes	Yes	Yes	Yes	Yes	Yes	Yes	Yes	Robust	Critically analyzed all aspects of the qualitative portion with adequate justifications. This is a good digital intervention for AYAs with cancer.
**Sansom-Daly**	Yes	Yes	Can't Tell	Yes	Yes	Yes	Yes	Can't Tell	Can't Tell	Yes	Moderate	The article does not adequately discuss the design, data analysis and findings. Some of the information provided feels incoherent.
**Wurz**	Yes	Yes	Can't Tell	Yes	Yes	Yes	Yes	Yes	Yes	Yes	Moderately strong	It covers most of the CASP checklist and can be recommended or modified to inform other similar interventions.
