# Supplementary material for: The Experiences of Adolescents and Young Adults with Digital Supportive Care Interventions for Cancer: A Systematic Review of Qualitative Studies
Source: Cancers (Basel). 2025 Feb 21;17(5):736. doi: 10.3390/cancers17050736 (PMC11899503; doi:10.3390/cancers17050736)
Supplement: Supplementary file 1 [file cancers-17-00736-s001.zip › Supplementary Table S7 (GRADE- CERQual).pdf]

Supplementary Table S7: GRADE-CERQual Assessment of confidence

Review question

1. What are the experiences of Adolescents and Young Adults (AYAs) with digital supportive care interventions for cancer? 2. What are the AYAs' perceived intervention barriers, motivators, and facilitators? 3. What are the potential areas for intervention improvement?

Authors of the review

Mashiad Mostafa, Y. Sarah Chae, Kelcey A. Bland, Helen McTaggart-Cowan

Summarized review finding	References	GRADE-CERQual Assessment of confidence	Explanation of GRADE-CERQual Assessment
**1. POSITIVE EXPERIENCES WITH INTERVENTION ATTRIBUTES**			
Sub-Theme 1.1. Appropriate Content: AYAs with cancer desired age-appropriate and relevant digital information. Positive experiences were associated with AYAs' comprehension and receptivity to the intervention's content.	Fergus et al. 2014; Hanghøj et al. 2023; Price & Brunet 2021; Wurz et al. 2023; Jibb et al. 2017; Melton et al. 2017; Miropolsky et al. 2020; Perumbil Pathrose et al. 2022; Poort et al. 2021; Heiniger et al. 2017; Markwardt et al. 2022; Mendoza et al. 2017; Phillips et al. 2023; Price & Brunet 2022;	**Moderate confidence**	No/Very minor concerns regarding coherence and relevance; Minor concerns regarding adequacy; Moderate concerns regarding methodological limitations
Sub-Theme 1.2. Flexible Choice: By having the option to personalize the interventions, participants can adapt the interventions to meet their self-identified needs. Examples of personalization include options to	Barnes et al. 2023; Donovan et al. 2019; Erickson et al. 2019; Fergus et al. 2014; Conduit et al. 2022; Hanghøj et al. 2023; Price & Brunet 2021; Lichiello et al. 2022; Wurz et al. 2023; Jibb et al. 2017; Miropolsky et al. 2020; Perumbil	**High confidence**	No/Very minor concerns regarding coherence, adequacy, and relevance; Moderate concerns

Supplementary Table S7: GRADE-CERQual Assessment of confidence

Summarized review finding	References	GRADE-CERQual Assessment of confidence	Explanation of GRADE-CERQual Assessment
select convenient dates and times for activity-related interventions and more	Pathrose et al. 2022; Poort et al. 2021; Sansom-Daly et al. 2019; Greer et al. 2019; Heiniger et al. 2017; Markwardt et al. 2022; Phillips et al. 2023; Micaux Obol et al. 2020;		regarding methodological limitations
Sub-Theme 1.3. Seamless Technology: AYAs preferred supportive care interventions when the technology was “fairly intuitive” and easy to use and navigate.	Erickson et al. 2019; Fergus et al. 2014; Price & Brunet 2021; Jibb et al. 2017; Melton et al. 2017; Miropolsky et al. 2020; Perumbil Pathrose et al. 2022; Poort et al. 2021; Greer et al. 2019; Heiniger et al. 2017; Markwardt et al. 2022; Mendoza et al. 2017; Price & Brunet 2022;	**Moderate confidence**	No/Very minor concerns regarding coherence and relevance; Minor concerns regarding adequacy; Moderate concerns regarding methodological limitations,
Sub-Theme 1.4. Inclusive Environment: AYAs with cancer expressed their preference for environments that they perceived to be inclusive. For some, favourable environments promoted psychological safety and enabled the AYAs to share their anxieties and thoughts with their peers.	Donovan et al. 2019; Hanghøj et al. 2023; Wurz et al. 2023; Jibb et al. 2017; Perumbil Pathrose et al. 2022; Sansom-Daly et al. 2019; Greer et al. 2019; Phillips et al. 2023; Price & Brunet 2022;	**Moderate confidence**	No/Very minor concerns regarding methodological limitations; Minor concerns regarding coherence, adequacy, and relevance

Supplementary Table S7: GRADE-CERQual Assessment of confidence

Summarized review finding	References	GRADE-CERQual Assessment of confidence	Explanation of GRADE-CERQual Assessment
**2. FACILITATED INTERVENTION OUTCOMES: HEALTH & WELL-BEING**			
Sub-Theme 2.1. Enhanced Connections and Communication Skills: Participants valued digital supportive care interventions that promoted a sense of community through their design elements; some examples include support groups, peer videos, and communication forums. Age- and cancer-appropriate content facilitated connection building with peers and family members, further enhancing well-being; for instance, participants felt they could relate to real-life survivor stories.	Barnes et al. 2023; Donovan et al. 2019; Erickson et al. 2019; Fergus et al. 2014; Conduit et al. 2022; Hanghøj et al. 2023; Price & Brunet 2021; Lichiello et al. 2022; Wurz et al. 2023; Jibb et al. 2017; Melton et al. 2017; Miropolsky et al. 2020; Poort et al. 2021; Sansom-Daly et al. 2019; Greer et al. 2019; Heiniger et al. 2017; Markwardt et al. 2022; Mendoza et al. 2017; Phillips et al. 2023; Price & Brunet 2022; Micaux Obol et al. 2020; Fergus et al. 2017;	**High confidence**	No/Very minor concerns regarding coherence, adequacy, and relevance Minor concerns regarding methodological limitations
Sub-Theme 2.2. Improved Physical Health: The provision of choice facilitated the set-up of personal goals and tracking of fitness metrics (e.g., sleep, activities, and calories). An inclusive environment was created through friendly competitions and	Barnes et al. 2023; Donovan et al. 2019; Erickson et al. 2019; Conduit et al. 2022; Hanghøj et al. 2023; Price & Brunet 2021; Wurz et al. 2023; Jibb et al. 2017; Miropolsky et al. 2020; Poort et al. 2021; Sansom-Daly et al. 2019; Heiniger et al. 2017; Markwardt et al.	**Moderate confidence**	No/Very minor concerns regarding coherence and relevance; Minor concerns regarding methodological limitations and adequacy

Supplementary Table S7: GRADE-CERQual Assessment of confidence

Summarized review finding	References	GRADE-CERQual Assessment of confidence	Explanation of GRADE-CERQual Assessment
reward systems that motivated participants to "get out and exercise" and establish healthy exercise and nutrition routines.	2022; Mendoza et al. 2017; Price & Brunet 2022; Micaux Obol et al. 2020;		
Sub-Theme 2.3. Improved Psychological Health: Overall, AYAs' experiences with the psychosocial supportive care interventions were positive. Improvements in their psychological health were observed, especially when the interventions contained appropriate content addressing positive psychology, such as cognitive, behavioral and mindfulness practices, music therapies, and coping approaches.	Barnes et al. 2023; Donovan et al. 2019; Erickson et al. 2019; Fergus et al. 2014; Conduit et al. 2022; Hanghøj et al. 2023; Price & Brunet 2021; Lichiello et al. 2022; Wurz et al. 2023; Jibb et al. 2017; Miropolsky et al. 2020; Perumbil Pathrose et al. 2022; Poort et al. 2021; Sansom-Daly et al. 2019; Greer et al. 2019; Heiniger et al. 2017; Phillips et al. 2023; Price & Brunet 2022; Micaux Obol et al. 2020; Fergus et al. 2017;	**High confidence**	No/Very minor concerns regarding methodological limitations, coherence, adequacy, and relevance
Sub-Theme 2.4. Autonomy: The positive contribution of the digital supportive care interventions in enhancing participants' health, communication, and connection enabled them to make autonomous	Barnes et al. 2023; Erickson et al. 2019; Fergus et al. 2014; Conduit et al. 2022; Hanghøj et al. 2023; Price & Brunet 2021; Lichiello et al. 2022; Jibb et al. 2017; Miropolsky et al. 2020; Greer et al. 2019; Heiniger et al. 2017;	**Moderate confidence**	No/Very minor concerns regarding coherence and relevance; Minor concerns regarding adequacy and

Supplementary Table S7: GRADE-CERQual Assessment of confidence

Summarized review finding	References	GRADE-CERQual Assessment of confidence	Explanation of GRADE-CERQual Assessment
decisions about their bodies and health. The knowledge gained from cancer-specific modules gradually enabled participants to overcome the social stigma and embarrassment of seeking help for mental health.	Markwardt et al. 2022; Mendoza et al. 2017; Phillips et al. 2023; Price & Brunet 2022; Micaux Obol et al. 2020;		methodological limitations
**3. NEGATIVE EXPERIENCES WITH INTERVENTION ATTRIBUTES**			
Sub-Theme 3.1. Generic Content: Participants did not appreciate busy layouts and "impersonal" content regardless of its delivery mode. Participants' experiences were suboptimal when the content of the interventions did not align with their cancer type, stage, or specific needs.	Donovan et al. 2019; Fergus et al. 2014; Conduit et al. 2022; Hanghøj et al. 2023; Price & Brunet 2021; Wurz et al. 2023; Jibb et al. 2017; Miropolsky et al. 2020; Perumbil Pathrose et al. 2022; Poort et al. 2021; Greer et al. 2019; Heiniger et al. 2017; Markwardt et al. 2022; Phillips et al. 2023; Micaux Obol et al. 2020; Fergus et al. 2017;	**Moderate confidence**	No/Very minor concerns regarding coherence and relevance; Minor concerns regarding methodological limitations; Moderate concerns regarding adequacy
Sub-Theme 3.2. Limited Choices: Participants considered it unfavourable to log in daily, having repetitive notifications with no option to mute,	Donovan et al. 2019; Fergus et al. 2014; Hanghøj et al. 2023; Wurz et al. 2023; Jibb et al. 2017; Miropolsky et al. 2020; Perumbil Pathrose et al. 2022; Poort et al. 2021; Sansom-Daly et al. 2019; Greer et al. 2019; Heiniger et al. 2017;	**Moderate confidence**	No/Very minor concerns regarding coherence, adequacy, and relevance; Minor concerns regarding

Supplementary Table S7: GRADE-CERQual Assessment of confidence

Summarized review finding	References	GRADE-CERQual Assessment of confidence	Explanation of GRADE-CERQual Assessment
more options with social media platforms	Markwardt et al. 2022; Mendoza et al. 2017; Phillips et al. 2023; Micaux Obol et al. 2020;		methodological limitations
Sub-Theme 3.3. Faulty Technology: Technological barriers—software glitches, time-outs, and battery issues—were observed to jeopardize the overall usability of the intervention.	Barnes et al. 2023; Fergus et al. 2014; Jibb et al. 2017; Miropolsky et al. 2020; Perumbil Pathrose et al. 2022; Sansom-Daly et al. 2019; Markwardt et al. 2022; Mendoza et al. 2017; Fergus et al. 2017;	**Moderate confidence**	No/Very minor concerns regarding coherence and relevance; Minor concerns regarding adequacy; Moderate concerns regarding methodological limitations
Sub-Theme 3.4. Unfavorable Environment: Very few participants expressed having too much anonymity felt counterproductive and desired more face-to-face interaction to feel included	Donovan et al. 2019; Hanghøj et al. 2023; Wurz et al. 2023; Miropolsky et al. 2020; Phillips et al. 2023;	**Low confidence**	Moderate concerns regarding methodological limitations, coherence and relevancy; Serious concerns regarding adequacy
**4. HINDERED INTERVENTION OUTCOMES: HEALTH AND WELL-BEING**			
Sub-Theme 4.1. Lack of Communication and Connection: Some participants were not able to engage	Donovan et al. 2019; Fergus et al. 2014; Conduit et al. 2022; Hanghøj et al. 2023; Wurz et al. 2023; Jibb et al. 2017;	**Moderate confidence**	No/Very minor concerns regarding coherence and relevance; Minor concerns

Supplementary Table S7: GRADE-CERQual Assessment of confidence

Summarized review finding	References	GRADE-CERQual Assessment of confidence	Explanation of GRADE-CERQual Assessment
their families to participate in interventions or form connections with the peer groups. Some AYAs associated anonymity with ingenuity and desired more face-to-face interaction [27,43] among participants and/or healthcare providers. Others felt the provision of all necessary cancer-related information left little room for queries and discussions, resulting in reduced community engagement.	Perumbil Pathrose et al. 2022; Poort et al. 2021; Greer et al. 2019; Mendoza et al. 2017; Phillips et al. 2023; Micaux Obol et al. 2020; Fergus et al. 2017;		regarding adequacy and methodological limitations
Sub-Theme 4.2. Triggering: Some AYAs experienced stressful emotions and felt overwhelmed by "…all sorts of gloomy things" brought forth by too much information. Some AYAs with cancer found it stressful to coordinate the virtual sessions. Not being able to meet exercise goals in physical activity-based interventions would demotivate participants, resulting in feelings of disappointment, stress and depression.	Donovan et al. 2019; Fergus et al. 2014; Hanghøj et al. 2023; Miropolsky et al. 2020; Poort et al. 2021; Sansom-Daly et al. 2019; Greer et al. 2019; Heiniger et al. 2017; Markwardt et al. 2022; Phillips et al. 2023; Price & Brunet 2022; Micaux Obol et al. 2020;	**Moderate confidence**	No/Very minor concerns regarding methodological limitations and relevance; Minor concerns regarding coherence; Moderate concerns regarding adequacy
